# Advances in predictive biomarkers associated with immunotherapy in extensive-stage small cell lung cancer

**DOI:** 10.1186/s13578-024-01283-9

**Published:** 2024-09-12

**Authors:** Tong Chen, Mingzhao Wang, Yanchao Chen, Yang Cao, Yutao Liu

**Affiliations:** Department of Medical Oncology, National Cancer Center/National Clinical Research Center for Cancer/Cancer Hospital, Chinese Academy of Medical Sciences and Peking Union Medical College, No. 17 Panjiayuan Nanli, Chaoyang District, Beijing, 100021 China

**Keywords:** Small cell lung cancer, Extensive stage, Immune checkpoint inhibitors, Immunotherapy, Efficacy, Predictive biomarkers

## Abstract

Small cell lung cancer (SCLC) is a highly malignant and poor-prognosis cancer, with most cases diagnosed at the extensive stage (ES). Amidst a landscape marked by limited progress in treatment modalities for ES-SCLC over the past few decades, the integration of immune checkpoint inhibitors (ICIs) with platinum-based chemotherapy has provided a milestone approach for improving prognosis, emerging as the new standard for initial therapy in ES-SCLC. However, only a minority of SCLC patients can benefit from ICIs, which frequently come with varying degrees of immune-related adverse events (irAEs). Therefore, it is crucial to investigate predictive biomarkers to screen potential beneficiaries of ICIs, mitigate the risk of side effects, and improve treatment precision. This review summarized potential biomarkers for predicting ICI response in ES-SCLC, with a primary focus on markers sourced from tumor tissue or peripheral blood samples. The former mainly included PD-L1 expression, tumor mutational burden (TMB), along with cellular or molecular components related to the tumor microenvironment (TME) and antigen presentation machinery (APM), molecular subtypes of SCLC, and inflammatory gene expression profiles. Circulating biomarkers predominantly comprised circulating tumor DNA (ctDNA), circulating tumor cells (CTCs), cytokines, plasma autoantibodies, inflammation-related parameters, and blood TMB. We synthesized and analyzed the research progress of these potential markers. Notably, investigations into PD-L1 expression and TMB have been the most extensive, exhibiting preliminary predictive efficacy in salvage immunotherapy; however, consistent conclusions have yet to be reached across studies. Additionally, novel predictive markers developed based on TME composition, APM, transcriptomic and genomic features provide promising tools for precision immunotherapy. Circulating biomarkers offer the advantages of convenience, non-invasiveness, and a comprehensive reflection of tumor molecular characteristics. They may serve as alternative options for predicting immunotherapy efficacy in SCLC. However, there is a scarcity of studies, and the significant heterogeneity in research findings warrants attention.

**Supplementary Information:**

The online version contains supplementary material available at 10.1186/s13578-024-01283-9.

## Background

Lung cancer is among the most prevalent malignant tumors globally and stands as the primary cause of cancer-related death. Lung cancer is histologically categorized into two major subtypes: non-small cell lung cancer (NSCLC) and small cell lung cancer (SCLC) [1]. SCLC is identified as a poorly differentiated neuroendocrine tumor, accounting for approximately 13–15% of all lung cancers [2]. SCLC is closely associated with tobacco exposure and is characterized by high malignancy, rapid growth, early distant metastasis, elevated recurrence rate, and acquired drug resistance [3], with a 5-year survival rate of less than 7% [4]. The Veterans Administration Lung Cancer Study Group (VALCSG) proposed dividing SCLC into limited-stage (LS) and extensive-stage (ES) disease, based on whether the lesions are contained to one hemithorax and can be covered by a radiation field [5]. Approximately 70% of newly diagnosed SCLC patients have already progressed to ES-SCLC. The standard first-line treatment for ES-SCLC using a platinum-etoposide (EP) combination has remained mostly unchanged for decades. ES-SCLC initially shows high sensitivity to chemotherapy, with a response rate of up to 60–65%, but the response is of short duration [6]. The median progression-free survival (PFS) spans only about 5–6 months, with the median overall survival (OS) of approximately 9–10 months [7, 8].

The clinical development of immunotherapy, especially anti-programmed cell death protein 1/programmed cell death 1 ligand 1 (PD-1/PD-L1) therapy, has been a revolutionary milestone in the treatment landscape of ES-SCLC in recent years. Landmark research such as IMpower133 and CASPIAN has supported the approval of atezolizumab/durvalumab plus chemotherapy for initial therapy of ES-SCLC globally. The IMpower133 study demonstrated that at a median follow-up of 13.9 months, the median OS for the atezolizumab group and the placebo group were 12.3 months and 10.3 months, respectively, with a hazard ratio (HR) of 0.70 (95% confidence interval [CI] 0.54–0.91; p = 0.007). Atezolizumab group had a median PFS of 5.2 months compared to 4.3 months for the placebo group, with a HR of 0.77 (95% CI 0.62–0.96; p = 0.02) [6]. The results of the CASPIAN study indicated that adding durvalumab to chemotherapy conferred a significant OS benefit compared to EP regimen (median OS: 12.9 months vs. 10.5 months; HR, 0.71; 95% CI 0.60–0.86; p = 0.0003) [9, 10]. Despite these advancements, the improvement in PFS and OS with the addition of immune checkpoint inhibitors (ICIs) is modest, and the divergence of long-term survival curves after six months suggested that a limited subset of SCLC patients benefited from ICIs. Additionally, while immunotherapy offers benefits, it also comes with immune-related toxicities [11–13]. Therefore, it is urgent to find reliable biomarkers to effectively predict the efficacy of PD-1/PD-L1 inhibitors.

Novel immunomodulatory agents beyond PD-1/PD-L1 inhibitors have also been intensively evaluated preclinically and clinically in SCLC, among which delta-like ligand 3 (DLL3)-targeted bispecific T-cell engagers (BiTEs) have garnered the most extensive research and demonstrated promising clinical efficacy [14–17]. Tarlatamab has received accelerated approval from the US Food and Drug Administration (FDA) for treating ES-SCLC following progression. Additionally, several other novel treatment strategies with promising preclinical results are undergoing corresponding clinical investigations, such as chimeric antigen receptor (CAR) based therapies [18–20], cancer vaccines [21, 22], and novel ICIs (anti-T cell immunoglobulin and mucin domain-containing protein 3 (TIM3), anti-T cell immunoreceptor with immunoglobulin and ITIM domain (TIGIT), anti-lymphocyte activation gene 3 (LAG3), anti‐CTLA4-LAG‐3 antibodies, etc.) [23–29]. However, the development of novel immunotherapies in SCLC largely remains in early-stage clinical trials, with limited exploration of biomarkers. The information of these trials was listed in Supplementary Table 1.

**Fig. 1:**
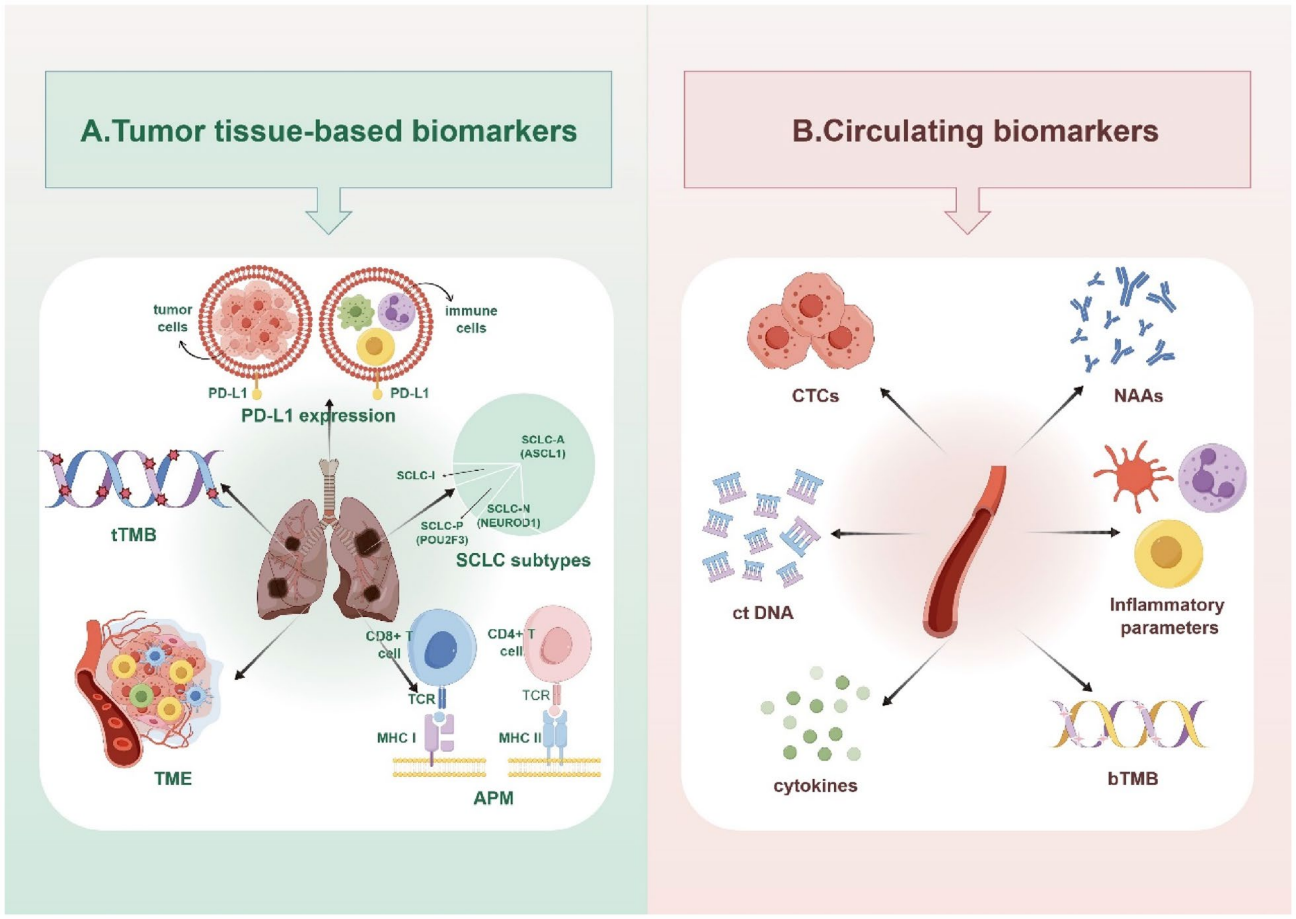
Predictive biomarkers of ICI response in ES-SCLC. SCLC: small cell lung cancer; ES: extensive stage; PD-L1: programmed cell death 1 ligand 1; ICI: immune checkpoint inhibitor; TMB: tumor mutational burden; tTMB: tissue tumor mutational burden; bTMB: blood tumor mutational burden; TME: tumor microenvironment; APM: antigen presentation machinery; TCR: T cell receptor; MHC: major histocompatibility complex; ASCL1: achaete-scute homologue 1; NEUROD1: neurogenic differentiation factor 1; POU2F3: POU class 2 homeobox 3; ctDNA: circulating tumor DNA; CTCs: circulating tumor cells; NAAs: neuronal autoantibodies

In this review, we aimed to summarize the latest advances in the predictive biomarkers for immunotherapy in ES-SCLC, with a primary focus on ICIs. Our primary emphasis, according to the types of markers that have been reported, lied on markers obtained from tumor tissue or peripheral blood (Fig. 1).

## Tumor tissue-based biomarkers

### PD-L1 expression

PD-L1 expression detected by immunohistochemistry (IHC) is considered a critical predictive factor for the immunotherapy response in NSCLC [30, 31]. Nonetheless, variations in PD-L1 expression levels in NSCLC arise from diverse clinical and genotypic characteristics among distinct study populations. Additionally, disparities in ICIs and corresponding detection platforms contribute to varying PD-L1 expression thresholds, resulting in inconsistent findings across studies [32]. In comparison to NSCLC patients, those with SCLC exhibit lower frequencies of PD-L1 expression, compounded by the scarcity of specimen cells which limits PD-L1 detection and research [33]. Reportedly, PD-L1 expression on tumor cells (TCs) in SCLC is quite low, with a range of 1.8% to 17%, whereas PD-L1 expression is more frequent on immune cells (ICs) compared to TCs, ranging from 25.8 to 40% [34–47]. Currently, the predictive role of PD-L1 expression in SCLC is still controversial (Table 1).

**Table 1:** PD-L1 expression as predictive biomarker for ICI therapies in clinical trials involving ES-SCLC

Trial	Phase	Year^a^	Population	Treatment	N (BEP)	Antibody
First-line setting						
CASPIAN [39]	III	2017	Untreated ES-SCLC	Durvalumab plus etoposide and platinum (EP) (D + EP), Durvalumab plus Tremelimumab plus EP (D + T + EP), EP alone	D + EP: 152 D + T + EP: 157 EP: 129	SP263
IMpower133 [34]	I/III	2016	Untreated ES-SCLC	Atezolizumab plus etoposide and carboplatin (EC), Placebo plus EC	Atezolizumab arm: 64, Placebo arm: 73	SP263
KEYNOTE-604 [36]	III	2017	Untreated ES-SCLC	Pembrolizumab plus EP, Placebo plus EP	Pembrolizumab arm: 185, Placebo arm: 175	22C3
ASTRUM-005 [37]	III	2019	Untreated ES-SCLC	Serplulimab plus EC, Placebo plus EC	Serplulimab arm: 379, Placebo arm: 186	22C3
CAPSTONE-1 [38]	III	2018	Untreated ES-SCLC	Adebrelimab plus EC, Placebo plus EC	Adebrelimab arm: 220, Placebo arm: 220	E1L3N
SKYSCRAPER-02 [25]	III	2020	Untreated ES-SCLC	Tiragolumab plus Atezolizumab and CE, Placebo plus Atezolizumab and CE	Tiragolumab arm: 216 Placebo arm: 225	SP263
First-line maintenance						
CheckMate 451 [40]	III	2015	ES-SCLC first line maintenance	Nivolumab plus Ipilimumab, Nivolumab monotherapy, Placebo	Combination arm: 116 Nivolumab arm: 124 Placebo arm: 114	28–8
NCT 02359019 [41]	II	2015	ES-SCLC first line maintenance	Pembrolizumab	TC assessed: 30, stroma interface assessed: 20	22C3
Second- and later-line settings						
CheckMate 331 [44]	III	2015	Relapsed ES-SCLC	Nivolumab, Chemotherapy (topotecan or amrubicin)	Nivolumab arm: 171 Chemotherapy arm: 150	28–8
KEYNOTE-158 [42]	II	2015	Relapsed ES-SCLC	Pembrolizumab	92	22C3
IFCT-1603 Trial [45]	II	2017	Relapsed SCLC	Atezolizumab, Chemotherapy (topotecan or re-induction of initial chemotherapy)	54	SP142
PASSION [46]	II	2018	Relapsed ES-SCLC	Camrelizumab plus apatinib	44	NR
CheckMate 032 [47]	I/II	2013	Relapsed SCLC	Nivolumab Nivolumab plus Ipilimumab	Nivolumab monotherapy: 78	28–8
KEYNOTE-028 [43]	Ib	2014	Relapsed ES-SCLC	Pembrolizumab	24	22C3

Trial	PD-L1 pattern	Cutoff	PD-L1 positivity	ORR%	mPFS (months)	mOS (months)
First-line setting						
CASPIAN [39]	ICs, TCs	1%	TC ≥ 1%: 5.7%, IC ≥ 1%: 25.8%, TC or IC ≥ 1%: 28.3%	D + EP vs. EP: ORs (95% CI) of 0.67 (0.06–5.87), 1.87 (0.64–5.88) and 1.68 (0.62–4.61), respectively, for the PD-L1 ≥ 1% subgroups based on TC, IC, and TC or IC expression; ORs of 2.26 (1.36–3.77), 2.21 (1.28–3.85), and 2.30 (1.32–4.07), respectively, for the PD-L1 < 1% subgroups; D + T + EP vs. EP: ORs of 3.89 (0.58–32.28), 0.82 (0.32–2.10) and 1.00 (0.40–2.42), respectively, for the PD-L1 ≥ 1% subgroups; ORs of 1.09 (0.67–1.77), 1.30 (0.75–2.25), and 1.22 (0.70–2.12), respectively, for the PD-L1 < 1% subgroups	D + EP vs. EP: HRs (95% CI) of 0.74 (0.22–2.24), 0.55 (0.32–0.96) and 0.55 (0.33–0.93), respectively, for the PD-L1 ≥ 1% subgroups based on TC, IC, and TC or IC expression; HRs of 0.69 (0.53–0.90), 0.74 (0.56–0.99), and 0.75 (0.56–1.00), respectively, for the PD-L1 < 1% subgroups; D + T + EP vs. EP: HRs of 0.13 (0.03–0.46), 0.66 (0.40–1.12) and 0.62 (0.38–1.01), respectively, for the PD-L1 ≥ 1% subgroups; HRs of 0.78 (0.60–1.01), 0.81 (0.60–1.08), and 0.83 (0.61–1.11), respectively, for the PD-L1 < 1% subgroups	D + EP vs. EP: HRs (95% CI) of 0.75 (0.24–2.27), 0.59 (0.34–1.02) and 0.61 (0.37–1.02), respectively, for the PD-L1 ≥ 1% subgroups based on TC, IC, and TC or IC expression; HRs of 0.62 (0.47–0.81), 0.64 (0.47–0.85), and 0.63 (0.47–0.85), respectively, for the PD-L1 < 1% subgroups; D + T + EP vs. EP: HRs of 0.42 (0.13–1.29), 0.53 (0.31–0.90) and 0.50 (0.31–0.83), respectively, for the PD-L1 ≥ 1% subgroups; HRs of 0.76 (0.58–0.99), 0.88 (0.66–1.19), and 0.91 (0.68–1.23), respectively, for the PD-L1 < 1% subgroups D + T + EP arm: TC or IC ≥ 1% subgroup vs. TC and IC < 1% subgroup: 15.5 vs. 8.9 (HR, 0.50; 95% CI 0.33–0.75)
IMpower133 [34]	TC or IC	1%, 5%	TC or IC ≥ 1%: 52.6%, TC or IC ≥ 5%: 21.2%	Atezolizumab arm vs. placebo arm: 75.0% vs. 62.2% in PD-L1 expression < 1% TC and IC (PD-L1 negative); 52.8% vs. 69.4% in PD-L1 expression ≥ 1% TC or IC	Atezolizumab arm vs. placebo arm: 5.4 vs. 4.2 (HR, 0.52; 95% CI 0.31–0.88) in PD-L1 negative; 5.1 vs. 5.5 (HR, 0.86; 95% CI 0.51–1.46) in PD-L1 expression ≥ 1% TC or IC	Atezolizumab arm vs. placebo arm: 10.2 vs. 8.3 (HR, 0.51; 95% CI 0.30–0.89) in PD-L1 negative; 9.7 vs. 10.6 (HR, 0.87; 95% CI 0.51–1.49) in PD-L1 expression ≥ 1% TC or IC; 21.6 vs. 9.2 (HR, 0.60; 95% CI 0.25–1.46) in PD-L1 expression ≥ 5% TC or IC;
KEYNOTE-604 [36]	CPS	1	40.8%	NR	Pembrolizumab arm vs. placebo arm: (HR, 0.73; 95% CI 0.54–1.01) in PD-L1 negative; (HR, 0.68; 95% CI 0.49–0.94) in PD-L1 CPS ≥ 1	Pembrolizumab arm vs. placebo arm: (HR, 0.80; 95% CI 0.58–1.11) in PD-L1 negative; (HR, 0.84; 95% CI 0.60–1.18) in PD-L1 CPS ≥ 1
ASTRUM-005 [37]	TPS	1%	17%	NR	NR	Serplulimab arm vs. placebo arm: 15.0 vs. 10.5 (HR, 0.58; 95% CI 0.44–0.76) in PD-L1 negative; not reached vs. 12.9 (HR, 0.92; 95% CI 0.44–1.89) in PD-L1 TPS ≥ 1%
CAPSTONE-1 [38]	TPS	1%	14%	NR	Adebrelimab arm vs. placebo arm: (HR, 0.68; 95% CI 0.54–0.85) in PD-L1 negative; (HR, 0.70; 95% CI 0.34–1.45) in PD-L1 positive	Adebrelimab arm vs. placebo arm: (HR, 0.66; 95% CI 0.52–0.83) in PD-L1 negative; (HR, 0.72; 95% CI 0.33–1.59) in PD-L1 positive
SKYSCRAPER-02 [25]	TC or IC	1%, 5%	TC or IC ≥ 1%: 58.0%, TC or IC ≥ 5%: 23.8%	NR	No significant difference reported (data not shown)	Tiragolumab arm vs. placebo arm: 10.8 vs. 12.0 (HR, 1.33; 95% CI 0.96–1.84) in PD-L1 < 1% TC and IC; 14.5 vs. 13.1 (HR, 1.01; 95% CI 0.75–1.37) in PD-L1 ≥ 1% TC or IC; 14.4 vs. 16.5 (HR, 1.56; 95% CI 0.97–2.51) in PD-L1 ≥ 5% TC or IC
First-line maintenance						
CheckMate 451 [40]	CPS	1%	46.0%	CPS ≥ 1% vs. CPS < 1%: 8.0% vs. 11.5% in combination arm, 9.8% vs. 13.2% in Nivolumab arm, 5.6% vs. 0% in placebo arm	CPS ≥ 1% vs. CPS < 1%: 2.8 (1.5–3.7) vs. 1.5 (1.4–2.5) in combination arm, 1.9 (1.4–4.1) vs. 1.6 (1.4–2.6) in Nivolumab arm, 1.4 (1.4–1.5) vs. 1.4 (1.4–1.5) in placebo arm	CPS ≥ 1% vs. CPS < 1%: 11.9 (6.9–15.2) vs. 8.6 (7.1–12.4) in combination arm, 14.1 (9.9–21.6) vs. 9.4 (5.8–11.3) in Nivolumab arm, 13.9 (8.9–16.5) vs. 6.1 (4.8–8.1) in placebo arm
NCT 02359019 [41]	TCs and stroma	1%	TCs: 10%, stroma interface: 40%	8.3% (95% CI 1.5–35.4) in stromal PD-L1 negative; 37.5% (95% CI 13.7–69.4) in stromal PD-L1 positive	1.3 (95% CI 0.6–2.5) in stromal PD-L1 negative; 6.5 (95% CI 1.1–12.8) in stromal PD-L1 positive	7.6 (95% CI 2.0–12.7) in stromal PD-L1 negative; 12.8 (95% CI 1.1–17.6) in stromal PD-L1 positive
Second- and later-line settings						
CheckMate 331 [44]	CPS	1	45.5%	NR	Nivolumab arm vs. chemotherapy arm: 1.4 vs. 4.1 (HR, 1.68; 95% CI 1.23–2.31) in PD-L1 negative; 1.5 vs. 4.3 (HR, 1.52; 95% CI 1.06–2.19) in PD-L1 CPS ≥ 1	Nivolumab arm vs. chemotherapy arm: 7.3 vs. 8.1 (HR, 0.91; 95% CI 0.66–1.25) in PD-L1 negative; 7.0 vs. 8.6 (HR, 0.96; 95% CI 0.67–1.38) in PD-L1 CPS ≥ 1
KEYNOTE-158 [42]	CPS	1	45.7%	6.0% in PD-L1 negative; 35.7% in PD-L1 positive	1.9 (95% CI 1.6–2.0) in PD-L1 negative; 2.1 (95% CI 2.0–9.9) in PD-L1 positive	7.7 (95% CI 3.9–10.4) in PD-L1 negative; 14.6 (95% CI 5.6–not estimable) in PD-L1 positive
IFCT-1603 Trial [45]	TCs, ICs, composite score	1% in ICs, 1% in TCs	TCs: 1.8%, ICs: 29.6%	6-week DCR in Atezolizumab arm: 0% in PD-L1 ≥ 1% IC; 25% in PD-L1 negative	PD-L1 negative vs. PD-L1 ≥ 1% IC: 1.4 (95% CI 1.2–1.5) vs. 1.6 (95% CI 1.4–4.1) (p = 0.15) in the whole BEP	PD-L1 negative vs. PD-L1 ≥ 1% IC: 5.6 (95% CI 2.4–9.5) vs. 12.0 (95% CI 4.1–13.3) (p = 0.39) in the whole BEP
PASSION [46]	NR	1%	23.4%	33.3% (95% CI 18.0–51.8) in PD-L1 negative; 45.5% (95% CI 16.7–76.6) in PD-L1 positive	3.7 (95% CI 1.2–4.6) in PD-L1 negative; 3.6 (95% CI 1.0–6.6) in PD-L1 positive	9.3 (95% CI 4.5–not reached) in PD-L1 negative; 6.6 (95% CI 1.9–12.3) in PD-L1 positive
CheckMate 032 [47]	TCs	1%	17%	Nivolumab monotherapy: 15.4% (95% CI 7.6–26.5%) in PD-L1 negative; 15.4% (95% CI 1.9–45.4%) in PD-L1 positive	NR	NR
KEYNOTE-028 [43]	TCs, ICs, stroma	1% of TCs and ICs or positive staining in stroma	only patients with PD-L1–positive tumors were enrolled	33.3% (95% CI 15.6%-55.3%)	1.9 (95% CI 1.7–5.9)	9.7 (95% CI 4.1-not reached)

PD-L1: programmed cell death 1 ligand 1; ICI: immune checkpoint inhibitor; SCLC: small cell lung cancer; ES: extensive stage; EC: etoposide and carboplatin; EP: etoposide and platinum; TCs: tumor cells; ICs: immune cells; CPS: combined positive score; TPS: tumor proportion score; ORR: objective response rate; DCR: disease control rate; mPFS: median progression-free survival; mOS: median overall survival; N: number; BEP: biomarker-evaluable population; HR: hazard ratio; CI: confidence interval; OR: odds ratio; NR: not reported

^a^“Year” refers to the first posted date in clinical trials

In the first-line treatment of ES-SCLC, the efficacy of ICIs combined with EP appears to be less dependent on PD-L1 expression. In the IMpower133 trial, OS benefits were observed with atezolizumab plus etoposide and carboplatin (EC) versus placebo plus EC in both the PD-L1 expression < 1% TC and IC subgroup (median OS: 10.2 months vs. 8.3 months; HR, 0.51; 95% CI 0.30–0.89) and the PD-L1 expression ≥ 5% TC or IC subgroup (median OS: 21.6 months vs. 9.2 months; HR, 0.60; 95% CI 0.25–1.46). Patients with PD-L1 expression levels ≥ 1% TC or IC, however, did not show a comparable outcome (median OS: 9.7 months vs. 10.6 months; HR, 0.87; 95% CI 0.51–1.49) [34, 35]. The KEYNOTE-604 study evaluated the efficacy of pembrolizumab plus EP in previously untreated ES-SCLC, revealing comparable HRs for PFS and OS between the PD-L1 combined positive score (CPS) ≥ 1 and PD-L1 negative subgroups [36]. The treatment regimens in the ASTRUM-005 trial [37] and CAPSTONE-1 trial [38] were respectively Serplulimab plus EC and Adebrelimab plus EC, and exploration of the predictive potential of PD-L1 expression yielded negative results consistent with previous studies. Interestingly, in exploratory analyses of the CASPIAN trial, OS benefit of durvalumab in combination with EP compared to EP alone appeared independent of PD-L1 expression, with HRs of 0.64 (95% CI 0.47–0.85) in the IC < 1% subgroup and 0.59 (95% CI 0.34–1.02) in the IC ≥ 1% subgroup. However, in the PD-L1 ≥ 1% subgroups, the OS benefit seemed greater with durvalumab plus tremelimumab plus EP versus EP alone, with HRs of 0.88 (95% CI 0.66–1.19) in the IC < 1% subgroup and 0.53 (95% CI 0.31–0.90) in the IC ≥ 1% subgroup, indicating that PD-L1 expression could potentially function as a promising biomarker for assessing the effectiveness of combination therapy involving PD-1/PD-L1 and cytotoxic T lymphocyte antigen 4 (CTLA-4) inhibition [39]. Whereas, further data from additional studies are required to bolster this proposition.

The available evidence on the predictive value of PD-L1 expression in first-line maintenance therapy for ES-SCLC remains insufficient. Exploratory analyses of the CheckMate451 study in the CPS-evaluable population demonstrated that PD-L1 expression levels (CPS ≥ 1 or < 1) were not associated with the benefits of nivolumab with or without ipilimumab compared to placebo as first-line maintenance therapy for ES-SCLC. However, across all treatment arms, including the placebo arm, patients with CPS ≥ 1 showed longer OS compared to patients with CPS < 1, indicating that PD-L1 expression might serve as a prognostic biomarker for ES-SCLC [40]. A phase II clinical trial assessed the effectiveness of maintenance pembrolizumab in ES-SCLC patients following chemotherapy. The findings indicated that the 8 patients with tumors positive for stromal PD-L1 expression achieved a higher median PFS (6.5 months vs. 1.3 months) and a higher median OS (12.8 months vs. 7.6 months) than the 12 patients with PD-L1-negative tumors, suggesting a potential benefit trend for pembrolizumab maintenance therapy in PD-L1 positive patients. However, the sample size of this study was limited (N = 20), and the results did not reach statistical significance [41].

In second- or later-line treatment for SCLC, the relationship between PD-L1 expression and the efficacy of ICIs has not reached a consensus. The KEYNOTE-158 trial, a phase II basket study of 11 cancer types, observed that pembrolizumab exhibited superior antitumor effects and sustained responses in ES-SCLC patients with PD-L1 CPS ≥ 1 compared to those who were PD-L1 negative, indicating that PD-L1 CPS could predict outcomes in ES-SCLC patients [42]. In the phase I, multicohort KEYNOTE-028 study, patients with PD-L1-positive recurrent or metastatic SCLC who received pembrolizumab monotherapy achieved an objective response rate (ORR) of up to 33.3%, with a median OS of 9.7 months (95% CI 4.1-not reached), indicating promising antitumor activity of pembrolizumab in PD-L1 positive SCLC patients [43]. Nonetheless, the pooled analysis of KEYNOTE-158 and KEYNOTE-028 explored the efficacy of pembrolizumab in recurrent SCLC patients who had undergone two or more lines of treatment. The results showed that pembrolizumab exhibited antitumor activity regardless of PD-L1 expression [48]. Likewise, in the CheckMate 331 study, the PD-L1 CPS status with a threshold of 1 did not impact the OS or PFS outcomes of nivolumab compared to chemotherapy [44]. Comparable findings were reported in the IFCT-1603 trial, which assessed the efficacy of atezolizumab as a second-line therapy for SCLC [45]. The PASSION study is a phase II trial of camrelizumab and apatinib in refractory ES-SCLC after platinum-based chemotherapy. The ORR (45.5% vs. 33.3%) was higher in the PD-L1-positive subgroup compared to the PD-L1-negative subgroup, but the median OS (6.6 months vs. 9.3 months) was shorter in patients with positive PD-L1, suggesting that the prognostic value of PD-L1 remained unvalidated [46]. The Phase I/II clinical trial CheckMate 032 evaluated the effectiveness of later-line nivolumab monotherapy or nivolumab plus ipilimumab, indicating that PD-L1 expression might not be a reliable indicator for the response to nivolumab [47].

In summary, the reliability of PD-L1 expression as a marker for immunotherapy response in ES-SCLC has not yet been supported by large-scale, high-quality randomized controlled trials (RCTs). The temporal and spatial heterogeneity of PD-L1 expression, variations in sensitivity among PD-L1 IHC detection antibodies, and the absence of standardized cutoff value for PD-L1 expression assessment may all impact its predictive value.

### Tissue tumor mutational burden (tTMB)

Tumor mutational burden (TMB) typically refers to the total count of somatic mutations per coding region of a tumor genome, as detected by whole exome sequencing (WES) or next generation sequencing (NGS) [49]. Based on the source of samples, it can be categorized into tissue TMB (tTMB) and blood TMB (bTMB). TMB serves as an indirect indicator of a tumor's capacity to produce neoantigens and has been shown to predict immunotherapy response across various cancer types, such as NSCLC, melanoma, and urothelial carcinoma, etc. [50–55] SCLC is marked by high TMB, possibly due to its strong association with smoking [56]. Nevertheless, the application of TMB in predicting ICIs response in SCLC remains contentious, given the heterogeneous outcomes observed across different studies (Table 2). Herein, our primary focus was on the findings related to tTMB, while analyses of bTMB were deliberated separately in the "Circulating biomarkers" section.

**Table 2:** Evidence of TMB as an immunotherapy-related biomarker in clinical trials involving ES-SCLC

TMB type	Trial	Phase	Year^a^	Population	Treatment	TMB assay
tTMB	First-line setting					
	CASPIAN [39]	III	2017	Untreated ES-SCLC	Durvalumab plus etoposide and platinum (EP) (D + EP), Durvalumab plus Tremelimumab plus EP (D + T + EP), EP alone	FoundationOne CDx
	KEYNOTE-604 [60]	III	2017	Untreated ES-SCLC	Pembrolizumab plus EP, Placebo plus EP	WES
	First-line maintenance					
	CheckMate 451 [40]	III	2015	ES-SCLC first line maintenance	Nivolumab plus Ipilimumab, Nivolumab monotherapy, Placebo	FoundationOne CDx
	Second- and later-line settings					
	CheckMate 331 [44]	III	2015	Relapsed ES-SCLC	Nivolumab, Chemotherapy (topotecan or amrubicin)	Foundation-One CDx
	KEYNOTE-158 [57]	II	2015	Relapsed ES-SCLC	Pembrolizumab	FoundationOne CDx
	CheckMate 032 [47, 51]	I/II	2013	Relapsed SCLC	Nivolumab, Nivolumab plus Ipilimumab	WES
bTMB	IMpower133 [34]	I/III	2016	Untreated ES-SCLC	Atezolizumab plus carboplatin and etoposide (EC), Placebo plus EC	NR

TMB type	Trial	Cutoff	N (BEP)	ORR%	mPFS (months)	mOS (months)
tTMB	First-line setting					
	CASPIAN [39]	6–14 mut/Mb	D + EP arm: 107, D + T + EP arm: 105, EP arm: 71	D + EP vs. EP: ORs (95% CI) of 1.98 (0.99–4.01), 1.85 (0.87–3.92), 1.99 (0.88–4.54), 1.59 (0.67–3.73), 1.23 (0.46–3.22), 0.97 (0.34–2.69), 1.14 (0.38–3.29), 0.75 (0.22–2.39) and 0.46 (0.10–1.78), respectively, for the tTMB-high subgroups at cutoffs ranging from 6 to 14 mut/Mb; ORs of 1.39 (0.32–6.36), 1.96 (0.60–6.71), 1.76 (0.66–4.79), 2.42 (0.95–6.36), 2.73 (1.17–6.59), 2.90 (1.29–6.72), 2.53 (1.16–5.67), 2.90 (1.36–6.31) and 2.86 (1.40–5.95) for the tTMB-low subgroups; D + T + EP vs. EP: ORs of 0.87 (0.43–1.75), 0.78 (0.36–1.64), 0.71 (0.31–1.61), 0.64 (0.27–1.49), 0.46 (0.17–1.22), 0.32 (0.11–0.90), 0.30 (0.09–0.91), 0.33 (0.09–1.07) and 0.30 (0.07–1.11) for the tTMB-high subgroups; ORs of 0.81 (0.24–2.76), 1.04 (0.37–2.94), 1.09 (0.44–2.70), 1.19 (0.50–2.85), 1.32 (0.60–2.90), 1.50 (0.70–3.26), 1.41 (0.67–2.96), 1.25 (0.61–2.57) and 1.18 (0.59–2.36) for the tTMB-low subgroups;	D + EP vs. EP: HRs (95% CI) of 0.75 (0.53–1.09), 0.83 (0.56–1.23), 0.76 (0.50–1.17), 0.76 (0.49–1.20), 0.79 (0.48–1.32), 0.82 (0.49–1.40), 0.79 (0.46–1.38), 0.79 (0.43–1.45) and 0.79 (0.39–1.62), respectively, for the tTMB-high subgroups at cutoffs ranging from 6 to 14 mut/Mb; HRs of 0.67 (0.30–1.43), 0.45 (0.23–0.85), 0.70 (0.42–1.15), 0.70 (0.44–1.12), 0.68 (0.44–1.05), 0.68 (0.45–1.02), 0.69 (0.46–1.04), 0.71 (0.48–1.04) and 0.70 (0.49–1.01) for the tTMB-low subgroups; D + T + EP vs. EP: HRs of 0.90 (0.62–1.32), 0.93 (0.62–1.40), 0.94 (0.61–1.47), 0.91 (0.57–1.45), 1.04 (0.63–1.75), 1.22 (0.73–2.11), 1.25 (0.72–2.21), 1.14 (0.63–2.12) and 1.24 (0.65–2.46) for the tTMB-high subgroups; HRs of 0.62 (0.33–1.20), 0.66 (0.38–1.16), 0.73 (0.45–1.18), 0.78 (0.49–1.23), 0.73 (0.48–1.11), 0.67 (0.44–1.02), 0.69 (0.47–1.04), 0.74 (0.51–1.10) and 0.74 (0.51–1.08) for the tTMB-low subgroups;	D + EP vs. EP: HRs (95% CI) of 0.72 (0.50–1.03), 0.80 (0.54–1.18), 0.71 (0.47–1.09), 0.72 (0.46–1.13), 0.68 (0.42–1.14), 0.69 (0.41–1.19), 0.65 (0.37–1.15), 0.66 (0.36–1.24) and 0.62 (0.30–1.32), respectively, for the tTMB-high subgroups at cutoffs ranging from 6 to 14 mut/Mb; HRs of 0.76 (0.34–1.66), 0.58 (0.31–1.09), 0.75 (0.45–1.26), 0.72 (0.45–1.18), 0.77 (0.50–1.20), 0.77 (0.50–1.17), 0.80 (0.53–1.20), 0.77 (0.52–1.13) and 0.76 (0.53–1.10), respectively, for the tTMB-low subgroups; D + T + EP vs. EP: HRs (95% CI) of 0.80 (0.55–1.17), 0.85 (0.56–1.28), 0.80 (0.52–1.25), 0.79 (0.50–1.26), 0.77 (0.46–1.30), 0.86 (0.51–1.49), 0.86 (0.49–1.54), 0.80 (0.43–1.52) and 0.86 (0.44–1.75), respectively, for the tTMB-high subgroups at cutoffs ranging from 6 to 14 mut/Mb; HRs of 0.84 (0.43–1.68), 0.76 (0.43–1.35), 0.81 (0.50–1.35), 0.82 (0.51–1.33), 0.83 (0.54–1.29), 0.78 (0.51–1.19), 0.79 (0.53–1.18), 0.82 (0.56–1.22) and 0.82 (0.56–1.20), respectively, for the tTMB-low subgroups;
	KEYNOTE-604 [60]	175 mut/exome	Pembrolizum arm: 167, Placebo arm: 151	NR	NR	Pembrolizum arm vs. placebo arm: 12.3 (8.3–15.5) vs. 12.0 (9.8–13.9) in TMB ≥ 175mut/exome (HR, 1.02; 95% CI 0.72–1.45); 10.2 (8.5–14.4) vs. 7.7 (6.6–9.3) in TMB < 175mut/exome (HR, 0.60; 95% CI 0.43–0.85)
	First-line maintenance					
	CheckMate 451 [40]	10, 13 mut/Mb	Nivolumab plus Ipilimumab: 192 Nivolumab: 196 Placebo: 192	Nivolumab plus Ipilimumab vs. Nivolumab vs. placebo: 14.0% vs. 14.3% vs. 5.3% in TMB ≥ 13mut/Mb; 6.5% vs. 9.5% vs. 4.0% in TMB < 13mut/Mb	Nivolumab plus Ipilimumab vs. Nivolumab vs. placebo: 2.7 (1.5–3.6) vs. 2.8 (1.6–4.1) vs. 1.6 (1.4–2.6) in TMB ≥ 13mut/Mb; 1.5 (1.4–2.0) vs. 1.6 (1.4–2.6) vs. 1.4 (1.4–1.4) in TMB < 13mut/Mb	Nivolumab plus Ipilimumab vs. placebo: 13.5 (9.3–21.8) vs. 9.5 (6.2–13.5) in TMB ≥ 13mut/Mb (HR, 0.61; 95% CI 0.39–0.94), 7.8 (6.7–9.7) vs. 10.0 (7.7–11.5) in TMB < 13mut/Mb (HR, 1.04; 95% CI 0.79–1.37); Nivolumab vs. placebo: 13.2 (10.0–17.9) vs. 9.5 (6.2–13.5) in TMB ≥ 13mut/Mb (HR, 0.67; 95% CI 0.45–1.01), 10.1 (8.7–11.3) vs. 10.0 (7.7–11.5) in TMB < 13mut/Mb (HR, 0.92; 95% CI 0.70–1.22)
	Second- and later-line settings					
	CheckMate 331 [44]	10, 11, 13, 14, 15 mut/Mb	Nivolumab arm: 155, Chemotherapy arm: 157	No significant difference reported (data not shown)	No significant difference reported (data not shown)	No significant difference reported (data not shown)
	KEYNOTE-158 [57]	10mut/Mb	SCLC cohort: 76	High TMB vs. non-high-TMB: 29.4% vs. 9.5%	No specific data of SCLC cohort; No significant difference reported in ten tumor-type-specific cohorts	High TMB vs. non-high-TMB: 9.4(5.6–19.1) vs. 6.3(3.9–7.7)
	CheckMate 032 [47, 51]	143, 247 mutations	Nivolumab arm:133, Nivolumab plus Ipilimumab arm: 78	Nivolumab arm vs. Nivolumab plus Ipilimumab arm: 21.3% vs. 46.2% in high TMB, 6.8% vs. 16.0% in medium TMB, 4.8% vs. 22.2% in low TMB	low TMB vs. medium TMB vs. high TMB: 1.3 (95% CI 1.2–1.4) vs. 1.3 (95% CI 1.2–1.4) vs. 1.4 (95% CI 1.3–2.7) in Nivolumab arm; 1.5 (95% CI 1.3–2.7) vs. 1.3 (95% CI 1.2–2.1) vs. 7.8 (95% CI 1.8–10.7) in Nivolumab plus Ipilimumab arm	low TMB vs. medium TMB vs. high TMB: 3.1 (95% CI 2.4–6.8) vs. 3.9 (95% CI 2.4–9.9) vs. 5.4 (95% CI 2.8–8.0) in Nivolumab arm; 3.4 (95% CI 2.8–7.3) vs. 3.6 (95% CI 1.8–7.7) vs. 22.0 (95% CI 8.2-not reached) in Nivolumab plus Ipilimumab arm
bTMB	IMpower133 [34]	10, 16 mut/Mb	346	NR	NR	Atezolizumab arm vs. placebo arm: 11.8 vs. 9.4 in bTMB < 10mut/Mb (HR, 0.73; 95% CI 0.49–1.08), 14.9 vs. 11.2 in bTMB ≥ 10mut/Mb (HR, 0.73; 95% CI 0.53–1.00); 12.5 vs. 10.0 in bTMB < 16mut/Mb (HR, 0.79; 95% CI 0.60–1.04), 17.1 vs. 11.9 in bTMB ≥ 16mut/Mb (HR, 0.58; 95% CI 0.34–0.99)

TMB: tumor mutational burden; tTMB: tissue TMB; bTMB: blood TMB; SCLC: small cell lung cancer; ES: extensive stage; N: number; BEP: biomarker-evaluable population; ORR: objective response rate; mPFS: median progression-free survival; mOS: median overall survival; EC: etoposide and carboplatin; EP: etoposide and platinum; mut/Mb: mutations per megabase; WES: whole exome sequencing; HR: hazard ratio; CI: confidence interval; NR: not reported; OR: odds ratio

^a^“Year” refers to the first posted date in clinical trials

The prospective biomarker analysis of the phase II KEYNOTE-158 study revealed that high tTMB was correlated with clinical benefit (ORR and OS) with pembrolizumab as later-line treatment in various tumor types, including SCLC [57]. The CheckMate 032 study [51] and CheckMate 451 study [40] evaluated the impact of TMB on the effectiveness of nivolumab alone or in combination with ipilimumab in the later-line treatment and maintenance therapy following first-line platinum-based chemotherapy for SCLC, respectively. The results of both studies suggested that TMB status could predict the response to these two treatment modalities. In the CheckMate 032 study, patients were stratified into low, medium, and high TMB tertiles on the basis of thresholds of 143 mutations and 247 mutations. It was reported that in both the monotherapy and combination therapy arms, patients with high TMB exhibited superior ORR, PFS, and OS than those with medium and low TMB [51]. In the CheckMate 451 study, OS was enhanced with both combination therapy (HR, 0.61; 95% CI 0.39–0.94) and monotherapy (HR, 0.67; 95% CI 0.45–1.01) compared to placebo in patients with TMB ≥ 13 mutations per megabase (mut/Mb) but not in the other patients [40]. However, the CheckMate 331 study assessed the correlation between high/low TMB and the effectiveness of later-line nivolumab using multiple cutoff values (10, 11, 13, 14, 15 mut/Mb), with results showing that TMB did not emerge as a predictor of clinical outcomes (p-value for interaction of TMB by treatment > 0.20 for all cutoffs) [44]. In conclusion, TMB holds promise as a predictive marker in ICI monotherapy or dual immunotherapy for previously treated advanced SCLC, and further validation is needed.

In other clinical settings, results may differ. Chemotherapy has the potential to elevate TMB, thereby complicating the assessment of the relationship between TMB and immunotherapy efficacy when combined with chemotherapy [58, 59]. Current clinical studies have yet to affirm the predictive capacity of TMB in first-line chemotherapy plus immunotherapy. The tTMB subgroups in the CASPIAN study were defined according to various tTMB thresholds ranging from 6 to 14 mut/Mb. Durvalumab in combination with EP or durvalumab plus tremelimumab plus EP showed consistent advantages over EP across these subgroups [39]. The phase III KEYNOTE-604 study in untreated ES-SCLC unveiled a positive correlation between high TMB and favorable OS in the placebo group (p = 0.005) but not in the pembrolizumab plus EP group (p = 0.450). Additionally, pembrolizumab plus EP demonstrated clinical benefit compared to placebo plus EP for TMB < 175mut/exome, but not for TMB ≥ 175 mut/exome [60]. Both studies have indicated that tTMB was not an ideal predictive biomarker.

According to current research, tTMB holds potential as a predictive marker for the efficacy of ICI monotherapy in later-line setting, but its role in first-line immunotherapy plus chemotherapy lacks supportive evidence. Furthermore, research on tTMB as a predictive biomarker is constrained by some limitations. The majority of existing studies are retrospective exploratory analyses with restricted sample sizes, along with a lack of standardized detection methods, platforms, and cutoff values. Therefore, further prospective studies with expanded sample sizes are warranted to clarify the prognostic role of tTMB in immunotherapies.

### Tumor microenvironment (TME)

The tumor microenvironment (TME) is a complex and dynamic network primarily comprised of tumor cells, immune cells (such as T lymphocytes, B lymphocytes, dendritic cells, and macrophages), stromal cells (including fibroblasts, endothelial cells, and pericytes), as well as various metabolites and cytokines [61]. The immune landscape within the TME exerts a pivotal influence on tumor initiation, progression, invasion, and resistance, thereby impacting patient prognosis [62, 63]. It has been reported that the TME of SCLC exhibited features of immune suppression, largely attributed to limited immune cell infiltration, low PD-L1 expression levels, and deficient antigen presentation [64–66]. Recent studies have explored TME-related predictive biomarkers to identify patients who may benefit from immunotherapy (Table 3). However, owing to the scarcity of both resected tumor samples and biopsy samples, such studies remain limited in number and are mostly retrospective in design.

**Table 3:** Other biomarkers for response or resistance to immunotherapy in ES-SCLC

Biomarker		Study	Author, year^a^	Population	Treatment	Assay
Tumor infiltrating lymphocytes, TILs		NCT02484404 (phase II trial) [70]	Thomas et al. 2015	Relapsed SCLC	Durvalumab and Olaparib	IHC
		CheckMate 032 (phase I/II trial) [68]	Rudin et al. 2013	Relapsed SCLC	Nivolumab, Nivolumab plus Ipilimumab	IHC
		A cohort study [69]	Shirasawa et al. 2023	Untreated SCLC	Atezolizumab plus carboplatin and etoposide (EC)	IHC
		A cohort study [71]	Pasello et al. 2023	Untreated ES-SCLC	Atezolizumab plus EC	9-color multiplex immunofluorescence
		A cohort study [72]	Kanemura et al. 2022	ES-SCLC	Chemotherapy plus an ICI (ICI combo-cohort); chemotherapy alone (chemo-cohort)	IHC
Molecular subtypes		CheckMate 032 (phase I/II trial) [68]	Rudin et al. 2013	Relapsed SCLC	Nivolumab alone, Nivolumab plus Ipilimumab	RNA sequencing
		KEYNOTE-604 (phase III trial) [60]	Rudin et al. 2017	Untreated ES-SCLC	Pembrolizumab plus EP, Placebo plus EP	RNA sequencing
		A cohort study [110]	Gay et al. 2021	Untreated ES-SCLC	Atezolizumab plus EC, Placebo plus EC	RNA sequencing
		IMpower133 (phase I/III trial) [111]	Liu et al. 2016	Untreated ES-SCLC	Atezolizumab plus EC, Placebo plus EC	RNA sequencing
		A cohort study [69]	Shirasawa et al. 2023	Untreated ES-SCLC	Atezolizumab plus EC	IHC
Antigen presentation machinery (APM)		CheckMate 032 (phase I/II trial) [68]	Rudin et al. 2013	Relapsed SCLC	Nivolumab alone, Nivolumab plus Ipilimumab	RNA sequencing
		CASPIAN (phase III trial) [105]	Garassino et al. 2017	Untreated ES-SCLC	Durvalumab plus Tremelimumab plus EP (D + T + EP), Durvalumab + EP (D + EP), EP	NGS
T cell-inflamed gene expression profile (TcellinfGEP)		KEYNOTE-604 (phase III trial) [60]	Rudin et al. 2017	Untreated ES-SCLC	Pembrolizumab plus EP, Placebo plus EP	RNA sequencing
		KEYNOTE-028 (phase Ib trial) [113]	Ott et al. 2014	Relapsed ES-SCLC	Pembrolizumab	RNA sequencing
RB1		A cohort study [117]	Dowlati et al. 2022	SCLC	Data set A: single-agent ICI ICI combination; Data set B (CheckMate 032) as validation: nivolumab, nivolumab plus ipilimumab	DNA and RNA sequencing
Circulating biomarkers	ctDNA	IFCT-1603 (phase II trial) [132]	Herbreteau et al. 2017	Relapsed SCLC	Atezolizumab, Chemotherapy (topotecan or re-induction of carboplatin-etoposide doublet)	NGS
		A cohort study [133]	Sivapalan et al. 2023	ES-SCLC	Immunotherapy-containing regimens, Chemotherapy	NGS
	Cytokines	NCT02484404 (phase II trial) [70]	Thomas et al. 2015	Relapsed SCLC	Durvalumab plus Olaparib	MSD U-PLEX
		A cohort study [166]	Hardy-Werbin et al. 2019	SCLC	EP (cohort 1), Ipilimumab plus EP (cohort 2)	Luminex assay
	neuronal auto-antibodies (NAAs)	NCT01331525 (phase II trial) [181]	Arriola et al. 2011	Untreated ES-SCLC	Ipilimumab plus EC	radioimmunoprecipitation assays, indirect IHC, immunoblotting, and a semiautomated enzyme-linked immunosorbent assay
		A cohort study [182]	Hardy-Werbin et al., 2018	Untreated SCLC	Cohort 1: EP, cohort 2: ipilimumab plus EC	Commercial immunoblotting assay
	NLR	A cohort study [192]	Xiong et al. 2021	Relapsed SCLC	ICIs	blood test
		A cohort study [193]	Riemann et al. 2023	Advanced SCLC	Atezolizumab plus EC	multi-color flow cytometry
		A cohort study [194]	Stratmann et al. 2022	Relapsed SCLC	Single-agent ICI ICI combination	blood test
	LIPI	A cohort study [195]	Li et al. 2021	Untreated advanced SCLC	ICIs plus chemotherapy	blood test

Biomarker		N (BEP)	ORR%	mPFS (months)	mOS (months)
Tumor infiltrating lymphocytes, TILs		14	Inflamed phenotype: tumor responses in all instances, immune-desert or immune-excluded phenotype: no tumor responses observed	NR	NR
		Nivolumab arm: 46, Nivolumab plus Ipilimumab arm: 32	NR	NR	CD8 positivity (≥ 1% infiltrating CD8 + T cells) vs. CD8 negative: (HR, 0.51; 95%CI 0.27–0.95) in Nivolumab arm; (HR, 0.70; 95%CI 0.32–1.49) in Nivolumab plus Ipilimumab arm
		37	NR	TIL-high (above median TIL density ≥ 69/mm2) vs. TIL-low: 7.3 (95% CI 4.2–10.4) vs. 4.0 (95% CI 2.7–5.3), p < 0.001	NR
		39	Correlated with better RR: lower CD163 + M2-polarized macrophages density and ratio on CD8 + cells in the total and tumoral areas (p < 0.05)	Correlated with longer PFS: lower CD163 + M2-polarized macrophages density and ratio on CD8 + cells (p < 0.05); high intra-tumoral CD4 + FOXP3 + density (p = 0.004); CD20 + interaction with CD8 + (p = 0.038); high percentage (%) of stromal CD163 + close to CD8 + cells (p = 0.045)	Correlated with longer OS: lower CD163 + M2-polarized macrophages density and ratio on CD8 + cells in the total and tumoral areas (p < 0.05); high intra-tumoral CD4 + FOXP3 + density (p = 0.026); CD20 + interaction with CD8 + (p = 0.032); high % of CD4 + closed to CD8 + cells in the total area (p = 0.025) and in the stroma (p = 0.002); intra and peri-tumoral interaction between CD163 + (p = 0.020) and CD8 + cells (p = 0.008); CD8 + and tumor cells interaction (p = 0.012)
		ICI combo-cohort: 63, chemo-cohort: 70	NR	Inflamed tumors (PD-L1-positive [CPS ≥ 1%] and CD8 + TIL-high [> 85/mm2]) vs. noninflamed tumors: 10.8 (95% CI 3.5–not reached) vs. 5.1 (4.3–5.6) (p = 0.002, HR, 0.26; 95% CI 0.09–0.74) in the ICI combo-cohort; 3.6 (95% CI 3.1–5.5) vs. 4.8 (4.4–5.7) (p = 0.11, HR, 1.70; 95% CI 0.92–3.14) in chemo-cohort	NR
Molecular subtypes		Nivolumab arm: 156, Nivolumab plus Ipilimumab arm: 130	NR	No significant difference reported (data not shown) based on subtype assignment (SCLC-A, -N, -P, and -Y)	No significant difference reported (data not shown)
		Pembrolizumab arm: 159, Placebo arm: 157	NR	NR	No significant difference reported (pembrolizumab arm, p = 0.960; placebo arm, p = 0.999) based on subtype assignment (SCLC-A, -N, -P, and -I)
		Atezolizumab arm: 132 Placebo arm: 139	NR	NR	Atezolizumab arm vs. placebo arm: (HR, 0.572; 95%CI 0.284–1.15) in SCLC-I, (HR, 0.807; 95%CI 0.547–1.189) in SCLC-A, (HR, 0.631; 95%CI 0.353–1.129) in SCLC-N, (HR,0.595; 95%CI 0.223–1.589) in SCLC-P; SCLC-I vs. all other tumors: (HR,0.566; 95%CI 0.321–0.998) in atezolizumab arm, (HR, 0.75; 95%CI 0.46–1.221) in placebo arm
		Atezolizumab arm: 122 Placebo arm: 131	Proportion of SCLC-I in long-term survivors (LTS) vs. non-LTS: Atezolizumab arm: 28% vs. 11% Placebo arm: 32% vs. 18%		
		34	NR	Pathological SCLC-A (pSCLC-A) vs. pSCLC-N vs. pSCLC-P: 4.8 (95% CI 3.9–5.7) vs. 4.0 (0.8–7.2) vs. 4.2 (2.9–5.5) (p value NR)	NR
Antigen presentation machinery (APM)		Nivolumab arm: 170 Nivolumab plus Ipilimumab arm: 151	NR	NR	APM-high vs. APM-med vs. APM low: p = 3.2 × 10^−4^ (Cox proportional hazards model) in nivolumab arm; p = 0.19 in nivolumab plus ipilimumab arm LSD1-low vs. LSD1-med vs. LSD1-high: p = 0.035 in nivolumab arm; p = 0.02 in nivolumab plus ipilimumab arm EZH2-low vs. EZH2-med vs. EZH2-high: p = 0.076 in nivolumab arm; p = 0.27 in nivolumab plus ipilimumab arm
		D + T + EP arm: 142, D + EP arm: 143, EP arm: 129	NR	NR	HLA-DQB1*03:01-positive vs. HLA-DQB1*03:01-negative: 14.9 (95% CI 10.4–21.2) vs. 10.5 (7.6–12.9) (HR, 0.59; 95% CI 0.39–0.88) in D + T + EP arm; 14.7 (11.5–16.3) vs. 14.3 (9.4–17.2) (HR, 0.93; 95% CI 0.63–1.37) in D + EP arm; 9.7 (7.7–11.7) vs. 10.5 (8.9–11.3) (HR, 0.94; 95% CI 0.61–1.40) in EP arm;
T cell-inflamed gene expression profile (TcellinfGEP)		Pembrolizumab arm: 159, Placebo plus EP: 157	NR	NR	Pembrolizum arm vs. placebo arm: 8.5 (95% CI 8.1–12.5) vs. 7.9 (95% CI 6.5–9.8) (HR, 0.74; 95%CI 0.49–1.11) in Tcell_inf_GEP < 1st tertile; 13.1 (95% CI 9.7–17.5) vs. 10.6 (95% CI 8.2–12.9) (HR, 0.77; 95%CI 0.56–1.06) in Tcell_inf_GEP > 1st tertile; Tcell_inf_GEP < 1st tertile vs. Tcell_inf_GEP > 1st tertile: p = 0.003 in pembrolizum arm; p < 0.005 in placebo arm
		All tumors: 313, SCLC: 8	Higher Tcell_inf_GEP score was associated with ORR across all tumors evaluated (p value NR)	Higher Tcell_inf_GEP score was associated with longer PFS across all tumors evaluated (p value NR)	NR
RB1		Data set A: 42; Data set B: 155 in nivolumab monotherapy arm, 124 in nivolumab plus ipilimumab arm	NR	Data set A: RB1 wild-type (WT) vs. RB1 mutant: 18 (95% CI 7-not reached) vs. 3 (1.5–24.1) (p = 0.05) in the entire cohort; Data set B: RB1 mutant vs. RB1 WT: (HR, 1.20; 95% CI 0.85–1.69; p = 0.294) in nivolumab monotherapy arm	Data set A: RB1 WT vs. RB1 mutant: 23.1 (95% CI 9–37.5) vs. 5 (2.5–26) (p = 0.04) in the entire cohort; Data set B: RB1 mutant vs. RB1 WT: (HR, 1.46; 95% CI 1.02–2.01; p = 0.041) in nivolumab monotherapy arm; (HR, 1.03; 95% CI 0.68–1.69; p = 0.9) in nivolumab plus ipilimumab arm
Circulating biomarkers	ctDNA	Atezolizumab arm: 46, Chemotherapy arm: 22	6-week DCR: detectable circulating mutation vs. no detectable mutation: 29.5% vs. 58.8% (p = 0.030) in all patients; 64.3% vs. 71.4% (p = 0.672) in chemotherapy arm; 13.3% vs. 50% (p = 0.0145) in atezolizumab arm	Low ctDNA abundance vs. high ctDNA abundance: 1.6 (95% CI 1.3–3.1) vs. 1.3 (1.1–1.5) (HR_VAF≥median_ = 2.57; 95% CI 1.31–5.07; p = 0.0063) in patients with a detectable circulating mutation	Low ctDNA abundance vs. high ctDNA abundance (median relative abundance = 44.5%): 12 (95% CI 6.6–15.3) vs. 2.5 (1.4–8.1) (HR_VAF≥median_ = 5.09; 95% CI 2.13–12.16; p = 0.0002) in patients with a detectable circulating mutation; 12.5 vs. 2.1 (HR_VAF≥median_ = 8.11; 95% CI 2.20–29.91; p = 0.0017) in atezolizumab arm; 9.4 vs. 7.7 (HR_VAF≥median_ = 0.91; 95% CI 0.21–3.98, p = 0.8996) in chemotherapy arm
		ICIs arm: 17, Chemotherapy arm: 16	NR	Patients with sustained early ctDNA elimination vs. ctDNA elimination followed by recrudescence vs. ctDNA burden persisted: not reached vs. 6.18 vs. 1.74 (p < 0.0001)	Patients with sustained early ctDNA elimination (ctDNA molecular responses) vs. ctDNA elimination followed by recrudescence vs. ctDNA burden persisted: not reached vs. 12.35 vs. 6.48 (p = 0.0006); ctDNA molecular responses vs. radiographic assessments: predictive performance for OS at 12 months (AUC 78.1% vs. 73.3%), 36 months (AUC 80.8% vs. 71.8%), and 64 months (AUC 87.3% vs. 67.6%)
	Cytokines	18	No significant difference reported (data not shown)	NR	NR
		Cohort 1: 47, cohort 2: 37	NR	NR	Baseline high IL-8 level vs. low IL-8 level: 9.2 vs. 16.8 (p = 0.028) in cohort 1; 5.3 vs. 17 (p = 0.031) in cohort 2; baseline high IL-2 level vs. low IL-2 level: 12.2 vs. 12.6 (p = 0.273) in cohort 1; 30.5 vs. 8 (p = 0.015) in cohort 2; baseline high IL-6 level vs. low IL-6 level: 15 vs. 12.6 (p = 0.073) in cohort 1; 9.5 vs. 18.5 (p = 0.026) in cohort 2; baseline high TNFα level vs. low TNFα level: 14.1 vs. 12.6 (p = 0.222) in cohort 1; 7.8 vs. 18.5 (p = 0.004) in cohort 2; IL-4 increased ≥ 23% from baseline to response vs. IL-4 increased < 23%: 9.5 vs. 16.3 (p = 0.002) in cohort 1; IL-4 increased ≥ 32% vs. IL-4 increased < 32%: 18.5 vs. 8.8 (p = 0.042) in cohort 2
	neuronal auto-antibodies (NAAs)	38	ORR based on immune-related response criteria (irRC): Any positive autoantibody at baseline vs. negative autoantibody: 100% (14/14) vs. 73.7% (14/19) (p = 0.049)	PFS by irRC (irPFS): Any positive autoantibody at baseline vs. negative autoantibody: 8.8 (95%CI 5.1–10.7) vs. 7.3 (2.9–7.9, p = 0.036); positive antinuclear antibody vs. negative antinuclear antibody: 10.2 vs. 6.9 (p = 0.032)	Any positive autoantibody vs. negative autoantibody: 18.5 vs. 17 (p = 0.144)
		Cohort 1: 47, cohort 2: 38	NAAs + (patients with at least 1 baseline positive NAA) vs. NAAs-: 96% vs. 91% (p = 0.451) in cohort 1; 90% vs. 93% (p = 0.635) in cohort 2	NAAs + vs. NAAs-: 7.4 (95% CI 6.2–8.6) vs. 6.2 (5.5–7) (p = 0.279) in cohort 1; 6.9 (6.6–7.2) vs. 6.9 (2.7–11.1) (p = 0.762) in cohort 2; The presence of only 1 NAA vs. > 1 NAA: 7.3 (5.9–8.6) vs. 5.5 (2.8–8.2) (p = 0.005) in cohort 2; Positive anti-Yo vs. negative anti-Yo: 4.3 (2.5–6.0) vs. 6.9 (6.4–7.5) in cohort 2	NAAs + vs. NAAs-: 15.1 (95%:CI 10.2–20) vs. 11.7 (7.5–15.9) (p = 0.032) in cohort 1; 12.3 (5.3–19.3) vs. 17.0 (0–36.1) (p = 0.796) in cohort 2; The presence of only 1 NAA vs. > 1 NAA: 12.3 (4.7–19.9) vs. 7.9 (4.9–11) (p = 0.180) in cohort 2; Positive anti-Yo vs. negative anti-Yo: 7.8 (2.2–13.4) vs. 17.0 (8.7–25.4) in cohort 2; patients with decreased or remained stable NAA during treatment vs. patients with increased NAA: 18.5 (15.8–21.2) vs. 12.3 (8.1–16.5) (p = 0.049) in both cohorts; 17.2 (12.4–22.1) vs. 12.6 (8.3–17) (p = 0.090) in cohort 1; 18.5 (9.1–27.8) vs. 9.5 (7.0–12.0) (p = 0.143) in cohort 2
	NLR	41	NLR < 5 vs. NLR ≥ 5 at 6 weeks post treatment: 67.9% vs. 46.2%	NLR < 5 vs. NLR ≥ 5 at 6 weeks post treatment: not reached vs. 3.2 (HR, 0.29; 95%CI 0.09–0.96; p = 0.04); NLR < 5 vs. NLR ≥ 5 at baseline: not reached vs. 4.8 (HR, 0.75; 95% CI 0.24–2.26; p = 0.58); PLR < 169 vs. PLR ≥ 169 at baseline: not reached vs. 5.1 (HR, 0.67; 95% CI 0.25–1.80; p = 0.43); PLR < 169 vs. PLR ≥ 169 at 6 weeks post treatment: not reached vs. 5.1 (HR, 0.69; 95% CI 0.25–1.86; p = 0.46)	NR
		40	NR	NLR ≥ 6.1 vs. NLR < 6.1: 5.1 ± 0.77 vs. 12.3 ± 1.8 (p = 0.001) (HR, 3.04; 95%CI 1.46–6.54; p = 0.003)	NLR ≥ 6.1 vs. NLR < 6.1: 7.1 ± 1.0 vs. 14.0 ± 1.7 (p = 0.001) (HR, 3.18; 95%CI 1.45–6.99;p = 0.004);
		Single ICI arm: 64 ICI combination arm: 47	No significant difference reported (data not shown)	NR	NLR ≥ median vs. NLR < median: 3.5 (95% CI 2.6–4.4) vs. 12.4 (3.6–21.3) (HR, 1.9; 95% CI 1.2–3.2; p = 0.008) in the whole cohort
	LIPI	100	NR	Pretreatment LIPI intermediate/poor vs. LIPI good (dNLR < 4.0 and LDH < 283U/L): 4.7 vs. 8.4 (p = 0.02) (multivariate analysis: HR, 1.42; 95% CI 0.84–2.39; p = 0.19)	Pretreatment LIPI intermediate/poor vs. LIPI good: 13.3 vs. 23.8 (p = 0.0006) (multivariate analysis: HR, 2.34; 95% CI 1.13–4.86; p = 0.02)

SCLC: small cell lung cancer; ES: extensive stage; N: number; BEP: biomarker-evaluable population; ORR: objective response rate; DCR: disease control rate; RR: response rate; mPFS: median progression-free survival; mOS: median overall survival; ICI: immune checkpoint inhibitor; PD-L1: programmed cell death 1 ligand 1; TILs: tumor infiltrating lymphocytes; FOXP3: forkhead box P3; APM: antigen presentation machinery; TcellinfGEP: T cell-inflamed gene expression profile; RB: retinoblastoma; WT: wild type; ctDNA: circulating tumor DNA; VAF: variant allele fraction; NAAs: neuronal autoantibodies; NLR: neutrophil-to-lymphocyte ratio; LIPI: lung immune prognostic index; LDH: lactate dehydrogenase; dNLR: derived neutrophils/ (leukocytes minus neutrophils) ratio; irRC: immune-related response criteria; EC: etoposide and carboplatin; EP: etoposide and platinum; CPS: combined positive score; IHC: immunohistochemistry; NGS: next generation sequencing; MSD: meso-scale discovery; LTS: long-term survivor; HR: hazard ratio; CI: confidence interval; NR: not reported

^a^“Year” refers to the first posted date in clinical trials, and to the date of publication in non-clinical-trial studies

It is widely believed that tumor infiltrating lymphocytes (TILs) within the TME serve multiple functions. TILs produce soluble cytokines that regulate tumor cell proliferation and metastasis, and directly participate in the immune-mediated anti-tumor mechanisms. Research has confirmed the correlations between TILs with superior prognosis in a variety of tumors, such as melanoma, colorectal cancer, and breast cancer [67]. Some studies have demonstrated that the degree of lymphocytes infiltration could predict ICIs response in ES-SCLC. For instance, the post hoc analysis of the CheckMate 032 study indicated that CD8 + T cell infiltration ≥ 1% was correlated with better survival in relapsed SCLC patients receiving nivolumab monotherapy (HR, 0.51; 95% CI 0.27–0.95), with a similar trend seen in patients receiving nivolumab plus ipilimumab (HR, 0.7; 95% CI 0.32–1.49) [68]. A retrospective study conducted by Shirasawa et al. validated the predictive value of TILs density in patients with treatment-naive ES-SCLC receiving atezolizumab plus EC [69]. Classification of immune phenotypes based on the presence and infiltration patterns of CD3 + and CD8 + lymphocytes has also been shown to predict their response to ICIs. An exploratory analysis of a single-arm phase II study revealed that tumors exhibiting an inflamed phenotype all experienced tumor remission following treatment with durvalumab combined with olaparib, while non-responding tumors displayed either an immune-desert or immune-excluded pattern [70]. Interestingly, Pasello et al. proposed a connection of immune cell distribution and their spatial indicators with the efficacy of first-line immunochemotherapy. Lower density of CD163 + M2 polarized macrophages and its ratio on CD8 + cells in both the overall and tumor regions were found to be favorably linked to PFS and OS (p < 0.05). Moreover, a high ratio of CD4 + to CD8 + cells adjacent in the entire region (p = 0.025) and stroma (p = 0.002), along with interaction between CD8 + cells and tumor cells (p = 0.012), were associated with longer OS. These findings highlighted the importance of the TME and cellular interactions in tumor response and survival prognosis [71]. Additionally, Kanemura et al. conducted a preliminary investigation into the potential of combining PD-L1 expression and TILs density as a prognostic indicator for ES-SCLC patients. They defined tumors with PD-L1 positivity (CPS ≥ 1%) and high CD8 + TILs (> 85/mm^2^) as “inflamed tumors,” while others were categorized as “non-inflamed tumors.” In the ICI plus chemotherapy cohort, median PFS for patients with inflamed tumors and non-inflamed tumors were 10.8 months (95% CI 3.5-not reached) and 5.1 months (95% CI 4.3–5.6), respectively (p = 0.002, HR, 0.26; 95% CI 0.09–0.74), indicating the predictive value of this combined biomarker [72].

Regulatory T cells (Tregs) expressing transcription factor forkhead box P3 (FOXP3) are crucial for maintaining dominant self-tolerance and immune homeostasis, typically inhibiting anti-tumor immune reactions and supporting tumor progression. However, FOXP3-TILs represent a heterogeneous population, comprising not only suppressive subsets but also non-suppressive subsets with anti-tumor activity [73, 74]. Two retrospective studies, involving 102 cases and 66 cases, respectively, have reported that FOXP3 + cells infiltration had an independently positive prognostic impact on patients with stages I to III SCLC [75, 76]. Unfortunately, immunotherapy was not included in the treatment modalities for these patients. Further research is needed to investigate the predictive value of FOXP3 + cells for immunotherapy response.

Tumor-associated macrophages (TAMs), a crucial component of the TME, can be generally categorized into anti-tumor M1 phenotype and pro-tumor M2 phenotype. The activity and phenotypes of TAMs can be dynamically regulated by integrating signals within the TME [77, 78]. Most clinical studies have observed that TAM infiltration was associated with the M2-phenotype-related gene expressions in solid tumors, where M2-like TAMs promoted angiogenesis and induced immune suppression [79–81]. However, there were also studies suggesting that macrophage infiltration might confer benefits to patients with solid tumors like NSCLC [82], colorectal cancer [83], and prostate cancer [84]. Eerola et al. evaluated samples from surgically treated SCLC patients, reporting that a higher concentration of macrophages was linked to better survival (p = 0.05) [85]. Another case–control study compared surgically resected tumor specimens from long-term SCLC survivors (survival > 4 years) and SCLC patients with expected survival time (survival < 2 years), revealing higher numbers of CD14 + monocytes, FOXP3 + lymphocytes, and CD68 + macrophages in long-term survivors (LTS). However, the relative counts of these cells in relation to CD3 + T lymphocytes were typically lower [86]. Both studies utilized surgical specimens and did not explore the correlation between macrophage infiltration and immunotherapy effect.

Chemokines exert a vital role in the migration of immune cells towards tumors, thereby modulating the immune landscape of the TME, usually favoring a pro-tumorigenic state [87]. Additionally, chemokines are involved in various cancer progression processes including cancer cell proliferation, tumor metastasis, angiogenesis, among others, thereby emerging as pivotal mediators of disease advancement with substantial implications for patient prognosis and treatment response [88, 89]. Chemokine (C–C motif) ligand 5 (CCL5), a member of the CC motif chemokine family, has been the subject of conflicting conclusions regarding its role in tumors. Some studies suggested that CCL5 served as an adverse prognostic indicator in cancer [90], while others proposed its protective role [91]. Tang et al. conducted a study using two published cohorts comprising 159 SCLC patients. Through the analysis of differentially expressed genes (DEGs) between high and low immune score, they observed a positive association between CCL5 expression with both survival and immunotherapy response in SCLC patients [92].

The exploration of TME-related biomarkers continues to encounter several challenges. Currently, most of the research remains exploratory and relies on retrospective data, lacking validation from RCTs. There is an urgent need to investigate standardized detection platforms. Furthermore, the constraints of single biomarkers underscore the necessity for developing composite predictive models that comprehensively reflect the immune status. Such an approach may act as an effective strategy for enhancing biomarker development.

### Antigen presentation machinery (APM)

The antigen presentation machinery (APM) is a crucial process for the correct identification, processing, and presentation of tumor antigens to CD8 + T cells, thereby triggering T cell immune-mediated cytotoxic killing [93]. Various factors that modify antigen display on tumor cells, such as genetic variations in genes encoding major histocompatibility complex (MHC) or other APM components, transcriptional and translational modulation, as well as epigenetic regulation, can impact the effectiveness of immune responses [94]. Thus, identifying the regulatory mechanisms of APM in tumors holds significant potential for the precise administration of immunotherapy.

MHC, also known as human leukocyte antigen (HLA), is a critical component of the APM, can be primarily divided into MHC class I and MHC class II molecules. The presentation of antigens by MHC class I molecules to CD8 + T cells is a key mechanism of immune surveillance [93]. Downregulation of MHC class I expression and subsequent decrease in antigen presentation contribute to immune escape by intracellular pathogens and malignant cells. SCLC exhibits poor immunogenicity, with most cases showing low expression or loss of MHC class I [95, 96]. A study has identified a specific subset of SCLC that exhibited high MHC I expression and displayed non-neuroendocrine features. Utilizing multiplexed immunofluorescence (mIF), spatial characterization in this subset revealed increased immune infiltration by CD3 + /CD8 + T cells and CD45 + /PD-L1 + immune cells, suggesting that the TME of such tumors might be poised for an anti-tumor response. Mahadevan et al. further corroborated a significant correlation between high MHC I expression and sustained clinical benefits from ICIs, indicating that MHC I could function as a marker for ICI response in SCLC [97]. Conversely, epigenetic silencing of MHC-I in SCLC leads to poor response to ICIs. A preclinical study conducted by Nguyen et al. illustrated that inhibition of lysine-specific demethylase 1 (LSD1) could restore cell surface expression of MHC-I, activate antigen presentation pathways, and enhance anti-tumor response to ICIs in SCLC [98].

MHC class II molecules are primarily expressed on professional antigen-presenting cells (APCs) and participate in the presentation of exogenous antigens to CD4 + T cells [99, 100]. Evidence suggested that HLA class II molecules on tumor cells influence tumor immunogenicity, tumor invasion, and immune responses [101, 102], while those on TILs are associated with antigen presentation, interactions with immune cells, and cancer prognosis [103]. In LS-SCLC patients, a retrospective study observed low expression of HLA class II on tumor cells while relatively high expression on TILs (positivity rates of 8.8% and 44.1% respectively). HLA class II on TILs was negatively correlated with lymph node metastasis and associated with longer recurrence-free survival (RFS), underscoring the prognostic and clinical significance of HLA class II in SCLC patients [104]. A post-hoc analysis of the phase III open-label CASPIAN study reported an association between the MHC class II allele DQB1*03:01 and longer OS in the durvalumab plus tremelimumab plus EP arm (HR, 0.59; 95%CI 0.39–0.88), but not in the durvalumab plus EP (HR, 0.93; 95%CI 0.63–1.37) or EP (HR, 0.94; 95%CI 0.61–1.40) arms [105].

The post-hoc analysis of the CheckMate 032 study preset a gene expression signature consisting of genes encoding the APM, such as HLA-A, HLA-B, HLA-C, B2M, TAP1, and TAP2. Rudin et al. assessed patient clinical outcomes by classifying cohorts of SCLC patients receiving nivolumab alone or with ipilimumab into tertiles based on APM gene signature. The results revealed a significant positive correlation (p = 3.2 × 10^−4^) between APM-related genes expression and OS for patients who received nivolumab. Furthermore, APM in SCLC is often subjected to epigenetic repression, with EZH2 and LSD1 identified as two critical negative epigenetic regulators. The study showed that elevated LSD1 expression was strongly linked to poorer OS in both the nivolumab and nivolumab plus ipilimumab arms (p = 0.035 and p = 0.02 respectively), with similar trends observed for EZH2 (p = 0.076 and p = 0.27 respectively) [68].

Research on the correlation of APM with benefit from ICIs in SCLC is still in its early stages (Table 3), necessitating further investigation and exploration.

### Molecular subtypes and gene expression profiling

As high-throughput sequencing technologies advance, whole-genome analysis of SCLC has revealed the complexity of its genomic landscape [106]. Research on the epigenetic and gene expression of preclinical models and human SCLC samples has identified distinct SCLC subtypes, uncovering significant heterogeneity within tumors, which correlated with tumor evolution, metastasis, and treatment resistance [107]. In 2019, Rudin et al. introduced a novel model of SCLC subtypes—A, N, P, and Y—defined by differential expression of four key transcription regulators: achaete-scute homologue 1 (ASCL1), neurogenic differentiation factor 1 (NEUROD1), POU class 2 homeobox 3 (POU2F3), and yes associated protein 1 (YAP1) [107, 108]. The first two are neuroendocrine subtypes, while the latter two are non-neuroendocrine subtypes. Diverse immune profiles exist among different SCLC subtypes, thus leading to varied benefits from immunotherapy. The exploratory analysis in the CheckMate 032 study investigated the relationship between these four subtypes and the survival benefits of ICIs. Unfortunately, statistical significance was not observed across all subtypes, but the APM gene signature was enriched in SCLC-Y (p < 10^–5^) [68]. Interestingly, Shirasawa proposed a pathological classification of SCLC on the basis of IHC evaluation of ASCL1, NEUROD1, POU2F3, and YAP1 expression: pathological SCLC-A (pSCLC-A), pSCLC-N, pSCLC-P, and pSCLC-Y. However, this retrospective study did not discover a connection between pathological subtypes and immunochemotherapy [69].

Nevertheless, subsequent IHC analyses failed to confirm a distinct TAP1-driven subtype [109]. Consequently, Gay et al. proposed a unique SCLC-I subtype, characterized by low expression of ASCL1, NEUROD1, and POU2F3, but with features of inflammatory genes and mesenchymal traits [110]. The research indicated that, compared to other subtypes, the SCLC-I subtype exhibited higher levels of CD8 + T cells, natural killer (NK) cells, macrophages, and B lymphocytes, along with increased expression of immune checkpoints and HLAs, illustrating superior responses to ICIs. The SCLC-I subtype was validated in tumor samples from IMpower-133 study. Although improvement trends were observed in the atezolizumab plus EC arm compared to the placebo plus EC arm across all four subtypes, the median OS and the magnitude of benefit with the addition of atezolizumab was numerically greater in SCLC-I (18.2 months vs. 10.4 months, HR, 0.57; 95% CI 0.28–1.15) compared to the other three subtypes. Additionally, the study noted a remarkable survival advantage of SCLC-I in OS over all other tumors in the atezolizumab plus EC arm (HR, 0.566; 95% CI 0.321–0.998) but not the placebo arm (HR, 0.75; 95%CI 0.46–1.221), suggesting that the SCLC-I subtype might be predictive of ICIs benefits [110]. Subsequently, exploratory analysis in IMpower-133 classified patients who survived for at least 18 months after randomization as LTS, evaluating the distribution of SCLC transcriptional subtypes in LTS and non-LTS groups. The results unveiled a greater percentage of LTS, especially in the atezolizumab group, with the SCLC-I subtype [111].

The 18-gene T cell–inflamed gene expression profile (TcellinfGEP) contains interferon (IFN)-γ-responsive genes linked to antigen presentation, chemokine expression, cytotoxic activity, and adaptive immune resistance, all crucial for clinical benefit [112]. TcellinfGEP has been developed into a clinical-grade assay and has been validated in some studies. For instance, the KEYNOTE-028 study, which encompassed patients with 20 distinct solid tumors including SCLC receiving pembrolizumab, revealed that patients achieving higher ORR and longer PFS had elevated TcellinfGEP scores. This underscored the predictive capability of TcellinfGEP for clinical benefits in PD-1 inhibitors [113]. However, this trial exclusively enrolled patients with PD-L1-positive solid tumors, thereby introducing bias in the distribution of biomarkers evaluated in the dataset, which posed limitations to its generalizability. Subsequent exploratory biomarker analyses in the KEYNOTE-604 study assessed the correlation of TcellinfGEP and SCLC transcriptional subtypes with survival outcomes. The findings indicated that SCLC subtypes were not linked to OS in either treatment group (pembrolizumab plus EP, p = 0.960; placebo plus EP, p = 0.999). However, a positive correlation between TcellinfGEP and OS was observed in both the pembrolizumab arm (p = 0.003) and the placebo arm (p < 0.005). Notably, there was no additional OS benefit with pembrolizumab plus EP [60].

The molecular hallmarks of SCLC encompass the inactivation of retinoblastoma gene (RB1), resulting in the absence of Rb protein expression, along with concomitant TP53 alterations [114]. SCLC exhibits near-universal biallelic functional inactivation of both RB1 and TP53 genes. RB1 is primarily involved in cell cycle regulation and cellular differentiation. Additionally, studies have highlighted the immunological significance of RB1, as evidenced by the downregulation of immune-related gene expression observed in preclinical models with RB1 inactivation [115, 116]. To assess the association between RB1 mutation or inactivation and the benefit of ICIs in SCLC, Dowlati et al. retrospectively collected data from 42 SCLC patients receiving either single-agent ICI or ICI combination therapy. They found that the median OS for patients with RB1 wild-type (WT) receiving ICI was 23.1 months (95% CI 9–37.5), compared to 5 months (95% CI 2.5–26; p = 0.04) for patients with RB1 mutation [117]. These results were further confirmed in CheckMate 032, where patients with RB1 mutant receiving nivolumab showed significantly inferior outcome compared to RB1 WT patients (HR, 1.41; 95% CI 1.02–2.01; p = 0.041). Moreover, a significant correlation was noted between a high RB1 loss-of-function signature score and the neuroendocrine subtype (ASCL1 and NEUROD1) [117].

In general, the development of predictive biomarkers for immunotherapy based on SCLC transcriptomic and genomic features is a promising field (Table 3). Such biomarkers hold the potential to guide the selection of more effective treatment strategies for SCLC patients. However, the role of molecular subtypes or inflammatory gene expression requires more RCTs to be substantiated.

## Circulating biomarkers

The conventional approach for clinical biomarker detection is tissue biopsy. However, this method presents certain limitations: (1) it is an invasive procedure; (2) tumors exhibit complex spatial and temporal heterogeneity, and a single biopsy may not encompass the full molecular characteristics of the tumor; (3) acquiring a sufficient quantity and quality of tumor specimens poses challenges [118]. In response to these constraints, liquid biopsy has gained growing prominence in recent years. Liquid biopsy primarily involves blood sampling but can also analyze cerebrospinal fluid, urine, pleural effusions, etc. It mainly detects circulating tumor DNA (ctDNA) and circulating tumor cells (CTCs) shed from primary or metastatic tumors into body fluids [119]. Liquid biopsy offers advantages such as low invasiveness, cost-effectiveness, and short detection time. It allows for repeated sampling to reflect tumor heterogeneity, as well as dynamic monitoring of treatment efficacy [120]. Liquid biopsy is often considered a rapid, minimally invasive alternative to tissue biopsy. In this chapter, we focused on the latest advancements in circulating biomarkers related to immunotherapy for SCLC (Table 3).

### Circulating tumor DNA (ctDNA)

ctDNA refers to specific DNA fragments released into the circulation either through active secretion of tumor cells or during tumor cell apoptosis or necrosis. ctDNA harbors genetic features derived from the tumor, such as gene mutations, methylation, copy number alterations (CNAs), etc. [121], serving as an important indicator for tumor screening [122], companion diagnostics [123], assessment of treatment efficacy and monitoring of recurrence [124–126]. Typically constituting a minor portion of cell-free DNA (cfDNA) in plasma, ctDNA can be identified using polymerase chain reaction (PCR) or NGS assays [127, 128].

Some studies have documented statistically significant correlations of quantified ctDNA variant allele fraction (VAF) and CNAs with OS, suggesting ctDNA as a prognostic biomarker for SCLC [129–131]. However, these studies did not include populations undergoing ICI therapy. Data on the predictive value of ctDNA in ES-SCLC patients receiving immunotherapy are limited.

According to an ancillary analysis of the phase II IFCT-1603 trial, high ctDNA abundance was significantly associated with poor OS outcomes (HR_VAF ≥median_, 8.11; 95% CI 2.20–29.91; p = 0.0017) in SCLC patients with atezolizumab as second-line treatment. Researchers observed that patients with high baseline ctDNA levels appeared to derive less benefit from atezolizumab than chemotherapy, while the reverse trend was observed in patients with low baseline ctDNA levels. This trial underscored the predictive role of ctDNA in second-line immunotherapy for SCLC [132].

Sivapalan et al. conducted a comprehensive longitudinal analysis of somatic sequence and plasma aneuploidy in ctDNA, identifying three distinct molecular response patterns reflecting different clinical outcomes in metastatic SCLC patients treated with either chemotherapy or immunotherapy-based regimens. Patients with sustained ctDNA elimination attained significantly longer OS (median OS: not reached) and PFS (median PFS: not reached) compared to those with ctDNA elimination followed by recrudescence (median OS: 12.35 months, median PFS: 6.18 months) or persistent ctDNA burden (median OS: 6.48 months, median PFS: 1.74 months) (p = 0.0006 and p < 0.0001, respectively). These findings suggested that longitudinal ctDNA dynamics assessment could provide a basis for early identification of persistent molecular response or resistance, guiding decisions to either continue or switch to alternative therapies for maximal clinical benefit [133, 134]. Similarly, in a phase II clinical trial evaluating the efficacy of durvalumab plus olaparib for relapsed SCLC, a case with a deleterious BRCA1 mutation was described, where the patient achieved a complete response (CR) accompanied by a sharp decline in cfDNA levels [70].

These studies supported the predictive significance of baseline and dynamic monitoring of ctDNA in SCLC patients undergoing immunotherapy, albeit with small cohorts. Prospective research is warranted to fully assess the reliability of ctDNA for clinical decision-making.

### Circulating tumor cells (CTCs)

CTCs are tumor cells that are shed from primary or metastatic sites into the peripheral blood. These cells carry vital information concerning the genetic and molecular characteristics of the tumor, facilitating real-time, dynamic, and non-invasive monitoring of the patient's condition [135]. They have shown prognostic significance across various cancer types, including breast cancer [136–138], NSCLC [139, 140], prostate cancer [141, 142], colorectal cancer [143, 144], and others.

Research confirmed that due to the short cell cycle and rapid proliferation of SCLC cells, which easily enter circulation leading to distant metastasis, the detection rate of CTCs in SCLC populations is approximately 60–94% [129, 145–150], significantly higher than in other tumors. Similar to ctDNA, studies on CTCs in SCLC primarily focused on their prognostic value, predominantly including SCLC cohorts treated with chemotherapy in the pre-immunotherapy era. Although specific thresholds have not been definitively established, these studies have documented a correlation between elevated levels of CTCs and unfavorable prognosis [146–153], with higher levels observed in ES-SCLC compared to LS-SCLC [146, 148, 150]. Furthermore, changes in CTC levels during treatment seem to predict clinical outcomes [149, 153, 154]. Additionally, the role of CTC detection in clinical disease differentiation [155], chemotherapy sensitivity evaluation [156, 157], and analysis of resistance molecular mechanisms [158] is supported by some research. The predictive potential of CTCs in immunotherapy remains to be further explored.

### Cytokines

Cytokines represent a class of soluble immune signaling proteins, including interleukins (IL), IFN, tumor necrosis factor (TNF), chemokines, and growth factors, which play pivotal roles in either promoting or inhibiting inflammation through various biochemical pathways and interactions [159, 160]. Preliminary data suggested that soluble factors such as IL-6 [161], IL-8 [162, 163], IL-10 [164, 165], etc., may serve as predictive or prognostic factors for ICI response in solid tumors such as NSCLC. However, there is limited research on the biological impact of cytokine levels in SCLC.

Hardy-Werbin and the team analyzed Th1, Th2, and proinflammatory cytokines in two independent cohorts of SCLC patients before and during treatment with chemotherapy with or without ipilimumab and correlated them with survival. The study noted an overall increase in all cytokines following treatment initiation in patients receiving ipilimumab. Irrespective of the treatment regimen, a high baseline IL-8 level was linked to poorer prognosis. Elevated baseline levels of IL-2 were indicative of sensitivity to ICIs, while high IL-6 and TNF-α predicted resistance. Additionally, an increase in IL-4 concentration during treatment in the immune-chemotherapy cohort correlated with improved OS [166]. However, a phase II clinical trial assessing the combination of durvalumab and olaparib for recurrent SCLC did not yield similar correlations [70]. Consequently, there remains no consensus regarding the role of cytokines in predicting ICI efficacy for SCLC, underscoring the need for further investigation.

### Serum neuronal autoantibodies (NAAs)

Paraneoplastic neurological syndromes (PNSs) are recognized as immune-mediated disorders, characterized by antibodies induced by tumor antigens that exhibit cross-reactivity with neural antigens [167]. Among patients with PNSs caused by SCLC, the most frequently detected onconeural autoantibodies are anti-Hu antibodies, also referred to as type 1 antineuronal nuclear antibodies (ANNA1) [168]. Additional onconeural autoantibodies implicated in PNSs include those targeting collapsin response mediator protein 5 (CRMP5), SOX1, microtubule-associated protein 1B (MAP1B), and amphiphysin [169]. PNSs manifest in 5–10% of SCLC patients, often accompanied by the detection of multiple autoantibodies. However, about half of patients without PNSs also carry at least one autoantibody [170–172].

In cases of PNSs related to SCLC, distinctive neurological dysfunction typically precedes respiratory symptoms, facilitating early cancer screening. Evidence suggested that SCLC patients with PNSs had a better prognosis than those without PNSs [173, 174]. Furthermore, several studies indicated potential prognostic value of certain neuronal autoantibodies (NAAs) such as ANNAs in SCLC [175, 176]. However, two other studies failed to observe prognostic differences between serum autoantibody-positive and -negative SCLC patients [170, 177].

Reportedly, SCLC patients with PNSs exhibit a "hot" TME marked by increased TILs, elevated PD-L1 expression, and increased PD-1/PD-L1 interactions, suggesting that such patients may represent an ideal population for receiving ICIs [178]. Additionally, immunotherapy can induce irAEs, and identifying serum characteristics before treatment commencement may help predict the risk of immune-mediated complications [179, 180].

In a biomarker analysis from a phase II clinical trial assessing ipilimumab plus EC as first-line treatment for ES-SCLC, autoimmune profile positivity at baseline was observed to be associated with improved outcomes and severe neurotoxicity [181]. Based on this study, Hardy-Werbin et al. expanded the research to include a control cohort receiving standard chemotherapy in order to evaluate the predictive and prognostic roles of NAAs. In both cohorts, the most prevalent autoantibody was anti-SOX1, succeeded by anti-HuD and anti-Yo. In the chemotherapy-alone cohort, positive NAAs at baseline correlated with better OS (15.1 months vs. 11.7 months, p = 0.032), whereas no such difference was observed in chemotherapy plus ipilimumab cohort (12.3 months vs. 17 months, p = 0.796). Furthermore, patients with a decrease in NAAs titer post-treatment experienced longer OS (18.5 months; 95%CI 15.8–21.2) compared to those with elevated NAAs (12.3 months; 95%CI 8.1–16.5; p = 0.049), indicating a correlation between antibody levels and tumor burden [182]. The findings demonstrated the function of NAAs as prognostic markers for SCLC and reflections of tumor burden, yet there was no conclusive evidence supporting their predictive role in ICI response. Further research is warranted to determine whether neuronal antibodies can serve as reliable predictors of immunotherapy efficacy and toxicity.

### Inflammatory hematologic parameters

Hematologic parameters, such as the neutrophil-to-lymphocyte ratio (NLR) and platelet-to-lymphocyte ratio (PLR), are described as general prognostic indicators for immunotherapy in several cancer types, reflecting the balance between pro-tumor inflammation and anti-tumor immune response [183–185]. Additionally, the lung immune prognostic index (LIPI), an index calculated from the lactate dehydrogenase (LDH) level and derived neutrophils/ (leukocytes minus neutrophils) ratio (dNLR), is believed to be linked with ICI outcomes in patients with melanoma and NSCLC [186–188].

The correlation of inflammation-related biomarkers with clinical outcomes in SCLC has been documented. NLR has been proposed as a significant prognostic marker for ES-SCLC patients across various treatments, excluding immunotherapy [189]. Sonehara et al. demonstrated LIPI as a prognostic factor for SCLC patients [190], a conclusion echoed by Qi et al. [191], although both studies involved patients who did not receive ICIs.

A retrospective study including 41 patients with SCLC who received anti-PD-1/PD-L1 antibodies as second- or later-line treatment evaluated NLR and PLR at baseline and 6 weeks post-treatment. Patients with NLR < 5 had significantly prolonged median PFS compared to those with NLR ≥ 5 at 6 weeks post treatment (HR, 0.29; 95%CI 0.09–0.96; p = 0.04), while a similar trend was not observed at baseline (HR, 0.75; 95% CI 0.24–2.26; p = 0.58), suggesting that the NLR at 6 weeks after start of treatment may predict early response in SCLC patients receiving ICIs [192]. Riemann et al. conducted an exploratory prospective study, identifying a high baseline NLR (NLR ≥ 6.1) as a risk factor for advanced SCLC patients’ response to chemotherapy combined with immunotherapy (median OS: HR, 3.18; 95%CI 1.45–6.99; p = 0.004) [193]. Similarly, Stratmann et al. proposed that NLR above the median was strongly correlated with inferior OS (3.5 months vs. 12.4 months; HR, 1.9; 95% CI 1.2–3.2; p = 0.008) in relapsed/refractory SCLC patients treated with ICIs [194]. A retrospective study explored the prognostic effect of LIPI on advanced SCLC patients undergoing first-line ICIs plus chemotherapy. The researchers found that the pretreatment LIPI good (dNLR < 4.0 and LDH < 283U/L) group had superior PFS (median: 8.4 months vs. 4.7 months, p = 0.02) and OS (median: 23.8 months vs. 13.3 months, p = 0.0006) than the LIPI intermediate/poor group, suggesting LIPI as a potential predictive biomarker [195]. Additional prospective research is required to evaluate the predictive capacity of these inflammatory markers for ICIs.

### Blood TMB (bTMB)

tTMB has been discussed in Section “Tumor tissue-based biomarkers” before. In contrast to tTMB, assessing bTMB may offer a more precise depiction of the overall disease characteristics, covering both primary and metastatic sites. Additionally, obtaining bTMB is more convenient and less invasive [196]. Retrospective analyses of the OAK and POPLAR studies have showcased consistency between bTMB and tTMB [197]. The correlation between bTMB and clinical outcomes of immunotherapy has been established in NSCLC [197–200]. Nevertheless, the predictive potential of bTMB in SCLC appears less promising. In the exploratory analysis of the IMpower133 trial, using 10 and 16 mut/Mb as bTMB thresholds, it was observed that atezolizumab plus EC exhibited enhanced efficacy over placebo plus EC, independent of bTMB levels [34]. Currently, there is a paucity of research exploring the predictive value of bTMB in SCLC patients with ICI monotherapy. Therefore, further investigation is warranted to elucidate the predictive role of bTMB on the efficacy of SCLC immunotherapy and its relationship with tTMB.

## Conclusions

The advent of immunotherapy has shown promise in improving outcomes for SCLC patients, although conferring benefits primarily to a small subset. Moreover, immunotherapy is accompanied by nearly unavoidable immune-related toxicities. Hence, there is a pressing clinical imperative to pinpoint suitable biomarkers to predict immunotherapy response so as to facilitate individualized treatment in SCLC. Conventional markers such as PD-L1 expression and TMB did not show consistent and robust predictive power for immunotherapy response in SCLC, though they played significant indicative roles in various cancer types. Notably, biomarkers based on TME, transcriptional and genetic characteristics may offer valuable guidance for immunotherapy. Specifically, TILs and RB1 mutation appear to hold promising predictive value. Moreover, given the advantages in convenience and reproducibility, circulating biomarkers, such as ctDNA, hold potential as alternative predictors of therapeutic efficacy. However, corresponding research data remains limited. Ultimately, owing to the pronounced heterogeneity of SCLC, the predictive utility of individual biomarker is constrained. The exploration of composite predictive models, integrating multi-omics information encompassing genomics, transcriptomics, proteomics, and epigenomics, may indeed represent a future trend.

## Supplementary Information

Supplementary Material 1.

## Data Availability

Not applicable.

## Abbreviations

SCLC: Small cell lung cancer
NSCLC: Non-small cell lung cancer
VALCSG: Veterans Administration Lung Cancer Study Group
ES: Extensive stage
LS: Limited stage
EP: Platinum and etoposide
PFS: Progression-free survival
OS: Overall survival
RFS: Recurrence-free survival
ICIs: Immune checkpoint inhibitors
HR: Hazard ratio
CI: Confidence interval
irAEs: Immune-related adverse events
PD-L1: Programmed cell death 1 ligand 1
PD-1: Programmed cell death protein 1
CAR: Chimeric antigen receptor
DLL3: Delta-like ligand 3
BiTEs: Bispecific T-cell engagers
FDA: The US Food and Drug Administration
TIM3: T cell immunoglobulin and mucin domain-containing protein 3
TIGIT: T cell immunoreceptor with immunoglobulin and ITIM domain
LAG3: Lymphocyte activation gene 3
IHC: Immunohistochemistry
TCs: Tumor cells
ICs: Immune cells
EC: Etoposide and carboplatin
CPS: Combined positive score
CTLA-4: Cytotoxic T lymphocyte antigen 4
CR: Complete response
ORR: Objective response rate
RCTs: Randomized controlled trials
TMB: Tumor mutational burden
tTMB: Tissue tumor mutational burden
bTMB: Blood tumor mutational burden
WES: Whole exome sequencing
NGS: Next generation sequencing
mut/Mb: Mutations per megabase
TME: Tumor microenvironment
TILs: Tumor infiltrating lymphocytes
Tregs: Regulatory T cells
FOXP3: Forkhead box P3
TAMs: Tumor-associated macrophages
LTS: Long-term survivor
CCL5: Chemokine (C–C motif) ligand 5
DEGs: Differentially expressed genes
APM: Antigen presentation machinery
MHC: Major histocompatibility complex
HLA: Human leukocyte antigen
mIF: Multiplexed immunofluorescence
LSD1: Lysine-specific demethylase 1
APCs: Antigen-presenting cells
ASCL1: Achaete-scute homologue 1
NEUROD1: Neurogenic differentiation factor 1
POU2F3: POU class 2 homeobox 3
YAP1: Yes associated protein 1
NK cells: Natural killer cells
TcellinfGEP: T cell–inflamed gene expression profile
RB1: Retinoblastoma
WT: Wild-type
ctDNA: Circulating tumor DNA
CNAs: Copy number alterations
VAF: Variant allele fraction
cfDNA: Cell-free DNA
CTCs: Circulating tumor cells
PCR: Polymerase chain reaction
IL: Interleukin
IFN: Interferon
TNF: Tumor necrosis factor
NAAs: Neuronal autoantibodies
PNSs: Paraneoplastic neurological syndromes
ANNA1: Type 1 antineuronal nuclear antibody
CRMP5: Collapsin response mediator protein 5
MAP1B: Microtubule-associated protein 1B
NLR: Neutrophil-to-lymphocyte ratio
PLR: Platelet-to-lymphocyte ratio
LIPI: Lung immune prognostic index
LDH: Lactate dehydrogenase
dNLR: Derived neutrophils/(leukocytes minus neutrophils) ratio
