# Supplementary material for: Advances in predictive biomarkers associated with immunotherapy in extensive-stage small cell lung cancer
Source: Cell Biosci. 2024 Sep 12;14:117. doi: 10.1186/s13578-024-01283-9 (PMC11391723; doi:10.1186/s13578-024-01283-9)
Supplement: Supplementary file 1 — Supplementary Material 1. [file 13578_2024_1283_MOESM1_ESM.docx]

Supplementary Table 1. Clinical trials of novel immunotherapies for SCLC

| Mechanism/  Class | Agent | Trial | Phase | Year* | Population | N | Status | Sponsor | Clinical efficacy | | Clinical safety |
| --- | --- | --- | --- | --- | --- | --- | --- | --- | --- | --- | --- |
| CAR therapies | | | | | | | | | | | |
| Anti-DLL3–transduced NK cells | DLL3-CAR-NK cells | NCT05507593 | I | 2022 | Relapsed ES-SCLC | 18 (estimated) | Recruiting | Tianjin Medical University Cancer Institute and Hospital | Unknown | | Unknown |
| Anti-DLL3–transduced autologous T cells | AMG 119 | NCT03392064(1) | I | 2018 | Relapsed SCLC | 5 | Suspended | Amgen Inc | One patient achieved a confirmed PR, two patients achieved SD, one patient had PD, and one patient’s response was unevaluable | | Reported TRAEs: pneumonitis, seizure, supraventricular tachycardia, and anemia |
|  | LB2102 | NCT05680922 | I | 2023 | Relapsed ES-SCLC and LCNEC | 41 (estimated) | Recruiting | Legend Biotech USA Inc | Unknown | | Unknown |
| T-cell engagers | | | | | | | | | | | |
| DLL3/CD3 BiTE | Tarlatamab (AMG757) | NCT03319940 (DeLLphi-300)(2) | I | 2017 | Relapsed SCLC | 107 | Recruiting | Amgen Inc | Confirmed ORR of 23.4% (including 2 [1.9%] complete and 23 [21.5%] partial responses);  DCR of 51.4% (95% CI, 41.5-61.2);  Median DoR of 12.3 months (95% CI, 6.6-14.9);  mPFS of 3.7 months (95% CI, 2.1-5.4) and mOS of 13.2 months (95% CI, 10.5-not reached) | TRAEs in 90.7% and grade ≥ 3 in 30.8% (grade 5 pneumonitis in 1%);  The most common TRAEs: CRS (52.3%), pyrexia (37.4%), dysgeusia (22.4%), fatigue (21.5%), and nausea (19.6%) | |
|  |  | NCT05060016 (DeLLphi-301)(3) | II | 2021 | Relapsed SCLC | 220 | Active, not recruiting | Amgen Inc | Objective response of 40% (97.5% CI, 29-52) in the 10mg group and 32% (97.5% CI, 21-44) in the 100mg group;  mPFS of 4.9 months (95% CI, 2.9-6.7) in the 10mg group and 3.9 months (95% CI, 2.6-4.4) in the 100mg group | The most common AEs: CRS (51% in the 10mg group and 61% in the 100mg group), decreased appetite (29% and 44%, respectively), and pyrexia (35% and 33%);  3% of the patients discontinued tarlatamab because of TRAEs | |
|  |  | NCT05740566 (Dellphi-304)(4) | III | 2023 | Relapsed SCLC | 490 (estimated) | Recruiting | Amgen Inc | Unknown | Unknown | |
|  | Tarlatamab (plus AMG 404) | NCT04885998 (DeLLphi-302) | I | 2021 | Relapsed SCLC | 23 | Active, not recruiting | Amgen Inc | Unknown | Unknown | |
|  | Tarlatamab (plus EC and PD-L1 inhibitor) | NCT05361395 (DeLLphi-303) | Ib | 2022 | Untreated ES-SCLC | 349 (estimated) | Recruiting | Amgen Inc | Unknown | Unknown | |
|  | Tarlatamab (plus Durvalumab) | NCT06211036 (DeLLphi-305) | III | 2024 | ES-SCLC first line maintenance | 550 (estimated) | Recruiting | Amgen Inc | Unknown | Unknown | |
|  | BI 764532 | NCT04429087(5) | I | 2020 | Refractory, DLL3-expressing SCLC and other NECs | 107 | Recruiting | Boehringer Ingelheim | ORR of 19% in all SCLC patients and 26% in SCLC patients who received ≥ 90μg/kg dose;  DCR of 39% in all SCLC patients and 51% in SCLC patients who received ≥ 90μg/kg dose; | The most common TRAEs (any/Grade 3+): CRS (48/2%), asthenia (32/3%), dysgeusia (27/0%), constipation (27/0%), lymphocyte count decreased (24/18%);  MTD has not been reached and dose escalation is ongoing;  DLTs were reversible and patients recovered | |
|  |  | NCT05882058 | II | 2023 | Relapsed SCLC and other NECs | 120 (estimated) | Recruiting | Boehringer Ingelheim | Unknown | Unknown | |
|  | BI 764532 (plus EP and anti-PD-L1 antibody) | NCT06077500(6) | I | 2023 | Untreated ES-SCLC | 60 (estimated) | Recruiting | Boehringer Ingelheim | Unknown | Unknown | |
|  | BI 764532 (plus Ezabenlimab) | NCT05879978 | I/II | 2023 | Refractory, DLL3-expressing SCLC and other NECs | 30 (estimated) | Recruiting | Boehringer Ingelheim | Unknown | Unknown | |
|  | BI 764532 (plus Topotecan) | NCT05990738 | I | 2023 | Relapsed ES-SCLC | 44 (estimated) | Recruiting | Boehringer Ingelheim | Unknown | Unknown | |
|  | QLS31904 | NCT05461287 | I | 2022 | Advanced solid tumors, including SCLC | 290 (estimated) | Recruiting | Qilu Pharmaceutical Co., Ltd | Unknown | Unknown | |
| Tri-specific recombinant protein construct | HPN328 | NCT04471727(7) | I/II | 2020 | Relapsed, advanced DLL3-expressing malignancies | SCLC: 10;  NE prostate cancer: 2;  other NE neoplasms: 4 | Recruiting | Harpoon Therapeutics | 3 of 9 (33%) SCLC patients had > 30% decrease in sum of target lesion diameters, including one confirmed PR;  2 of 4 (50%) patients treated at ≥ 1.215 mg/week had > 30% decrease | CRS transient and manageable, with 31% of patients experiencing grade 1-2 CRS; no grade ≥ 3 CRS reported;  No DLTs were observed, and no AEs led to discontinuation | |
| DLL3 x CD3/CD137 multispecific antibody | RO7616789 | NCT05619744 | I | 2023 | Relapsed ES-SCLC and other NECs | 168 (estimated) | Recruiting | Hoffmann-La Roche | Unknown | Unknown | |
| Anti-DLL3/CD47 bispecific antibody | PT217 | NCT05652686 | I/II | 2023 | SCLC and other NECs | 61 (estimated) | Recruiting | Phanes Therapeutics | Unknown | Unknown | |
| Anti‐GD2 × CD3 bispecific antibody | Nivatrotamab | NCT04750239 | I/ II | 2021 | Relapsed SCLC | 3 | Terminated | Y-mAbs Therapeutics | Unknown | Unknown | |
| Cancer vaccine | | | | | | | | | | | |
| mRNA-based neoantigen therapy | mRNA-4157 | NCT03313778 (KEYNOTE-603) | I | 2017 | resectable or unresectable solid tumors including SCLC | 242 (estimated) | Recruiting | ModernaTX, Inc. | Unknown | Unknown | |
| Novel immune-checkpoint inhibitors | | | | | | | | | | | |
| Anti-TIM3 and PD-1 bispecific  antibody | RO7121661 | NCT03708328 | I | 2018 | Advanced or metastatic solid tumors including SCLC | 134 (actual) | Active, not recruiting | Hoffmann-La Roche | Unknown | Unknown | |
| Anti-TIGIT antibody | Tiragolumab (plus Atezolizumab and EC) | NCT04256421 (SKYSCRAPER-02)(8) | III | 2020 | Untreated ES-SCLC | FAS (all patients): 490;  PAS (patients without brain metastases): 397 | Active, not recruiting | Hoffmann-La Roche | (Tiragolumab arm [Tiragolumab plus Atezolizumab and EC] vs. control arm [placebo plus Atezolizumab and EC])  In the PAS: mPFS: 5.4 vs. 5.6 months (HR, 1.11; 95% CI, 0.89-1.38; P = .3504); mOS: 13.1 vs. 13.1 months (HR, 1.14; 95% CI, 0.90-1.44; P = .2859);  In the FAS: mPFS: 5.1 vs. 5.4 months (HR, 1.08; 95% CI, 0.89-1.31); mOS: 12.8 vs. 12.9 months (HR, 1.09; 95% CI, 0.88-1.35; P = .4205) | (Tiragolumab arm vs. control arm)  Immune-mediated AEs: 54.4% vs. 49.2% (grade 3/4: 7.9% vs. 7.7%);  AEs leading to treatment withdrawal: 8.4% vs. 9.3% | |
|  | MK-7684A (Pembrolizumab/Vibostolimab co-formulation) | NCT05224141 (Keyvibe‐008) | III | 2022 | Untreated ES-SCLC | 450 (estimated) | Active, not recruiting | Merck Sharp & Dohme LLC | Unknown | Unknown | |
|  | IBI939 (plus Sintilimab) | NCT04672356 | I | 2020 | NSCLC and SCLC | 19 | Completed | Innovent Biologics (Suzhou) Co. Ltd. | Unknown | Unknown | |
| Anti-LAG3 antibody | LAG525 (plus PDR001) | NCT03365791(9) | II | 2017 | Advanced malignancies including SCLC | 76 | Completed | Novartis Pharmaceuticals | 24‐week clinical benefit rates of 0.27 in SCLC, meeting the primary endpoint | Unknown | |
|  | INCAGN02385 | NCT03538028(10) | I | 2018 | Advanced malignancies including SCLC | 22 | Completed | Incyte Biosciences International Sàrl | Unknown | Unknown | |
| anti‐CTLA4-LAG‐3 bispecific antibody | XmAb22841 (with or without Pembrolizumab) | NCT03849469 | I | 2019 | Advanced solid tumors including SCLC | 78 | Completed | Xencor, Inc. | Unknown | Unknown | |
| Other new targets | | | | | | | | | | | |
| Anti-GD2 antibody | Dinutuximab (plus irinotecan) | NCT03098030(11) | II/III | 2017 | Relapsed SCLC | 483 | Completed | United Therapeutics | (Dinutuximab/irinotecan arm vs. irinotecan arm vs. topotecan arm)  mOS: 6.9 vs. 7.0 vs. 7.4 months (p = 0.3132);  mPFS: 3.5 vs 3.0 vs 3.4 months (p = 0.3482);  ORR confirmed: 17.1% vs 18.9% vs 20.2% (p = 0.8043) | The most common toxicities: diarrhea (64.5%, 62.0%, and 14.8%), anemia (36.6%, 29.4%, and 65.9%), abdominal pain (44.8%, 12.8%, and 10.2%), and nausea (44.3%, 47.1%, and 25.0%) | |
| Fucosyl-GM1 monoclonal antibody | BMS-986012 (plus EC and Nivolumab) | NCT04702880 | II | 2021 | Untreated ES-SCLC | 120 (estimated) | Recruiting | Bristol-Myers Squibb | Unknown | Unknown | |
|  | BMS-986012 (with or without Nivolumab) | NCT02247349 (CA001-030)(12) | I/II | 2014 | Relapsed SCLC | 106 | Completed | Bristol-Myers Squibb | (BMS-986012 plus Nivolumab vs. BMS-986012)  ORR: 38% (95% CI, 20.7%–57.7%) vs. 4% (0.8%–11.0%)  PFS at 24 weeks: 39.3% (95% CI, 21.7%–56.5%) vs. 12.2% (6.0%–20.7%);  mPFS: 2.1 (95% CI, 1.4–9.9) vs. 1.3 (95% CI, 1.3–1.4) months  mOS: 18.7 (95% CI, 8.2–37.3) vs. 5.4 (4.0–7.3) months | The most common TRAE: pruritus (90%);  Grade 4 TRAEs in 2% of patients | |

**Abbreviations:** SCLC = small cell lung cancer; ES = extensive stage; LCNEC = large cell neuroendocrine lung carcinoma; NSCLC = non-small cell lung cancer; N = number; DLL3 = delta-like ligand 3; EP = etoposide and platinum; EC = etoposide and carboplatin; PD-L1 = programmed cell death 1 ligand 1; PD-1 = programmed cell death protein 1; CAR = chimeric antigen receptor; NK cell = natural killer cell; PR = partial response; SD = stable disease; PD = progressive disease; TRAEs = treatment-related adverse events; BiTE = bispecific T cell engager; CRS =cytokine release syndrome; NE = neuroendocrine; NEC = neuroendocrine carcinoma; ORR = objective response rate; DCR = disease control rate; DoR = duration of response; mPFS = median progression-free survival; mOS = median overall survival; HR = hazard ratio; CI = confidence interval; MTD = maximum tolerated dose; DLTs = dose-limiting toxicities; FAS = full analysis set; PAS = primary analysis set; TIGIT = T cell immunoreceptor with immunoglobulin and ITIM domain; TIM3 = T cell immunoglobulin and mucin domain-containing protein 3; LAG-3 = lymphocyte activation gene‐3; CTLA-4 = cytotoxic T-lymphocyte–associated antigen 4; GD2 = disialoganglioside;

* “Year” refers to the first posted date in clinical trials

# References

1. Byers LA, Heymach JV, Gibbons DL, Zhang J, Chiappori AA, Rasmussen ER, et al. 697 A phase 1 study of AMG 119, a DLL3-targeting, chimeric antigen receptor (CAR) T cell therapy, in relapsed/refractory small cell lung cancer (SCLC). Regular and Young Investigator Award Abstracts. 2022.

2. Paz-Ares L, Champiat S, Lai WV, Izumi H, Govindan R, Boyer M, et al. Tarlatamab, a First-in-Class DLL3-Targeted Bispecific T-Cell Engager, in Recurrent Small-Cell Lung Cancer: An Open-Label, Phase I Study. J Clin Oncol. 2023;41(16):2893-903.

3. Ahn MJ, Cho BC, Felip E, Korantzis I, Ohashi K, Majem M, et al. Tarlatamab for Patients with Previously Treated Small-Cell Lung Cancer. N Engl J Med. 2023;389(22):2063-75.

4. Paz-Ares LG, Felip E, Ahn M-J, Blackhall FH, Borghaei H, Cho BC, et al. Randomized phase 3 study of tarlatamab, a DLL3-targeting bispecific T-cell engager (BiTE), compared to standard of care in patients with relapsed small cell lung cancer (DeLLphi-304). Journal of Clinical Oncology. 2023;41(16_suppl):TPS8611-TPS.

5. Wermke M, Kuboki Y, Felip E, Alese OB, Morgensztern D, Sayehli C, et al. OA01.05 Phase I Dose Escalation Trial Of The DLL3/CD3 Igg-Like T Cell Engager BI 764532 In Patients with DLL3+ Tumors: Focus on SCLC. Journal of Thoracic Oncology. 2023;18(11):S45-S6.

6. Peters S, Champiat S, Yoshida T, Dorleacq N, Ma Y, Geng L, et al. DAREON-8: A phase I, open-label, dose escalation/expansion trial of the DLL3-targeting T-cell engager, BI 764532, combined with first-line (1L) standard of care (platinum, etoposide, and anti-PD-L1 antibody) in patients (pts) with extensive-stage small cell lung carcinoma (ES-SCLC). Journal of Clinical Oncology. 2024;42(16_suppl):TPS8127-TPS.

7. Johnson ML, Dy GK, Mamdani H, Dowlati A, Schoenfeld AJ, Pacheco JM, et al. Interim results of an ongoing phase 1/2a study of HPN328, a tri-specific, half-life extended, DLL3-targeting, T-cell engager, in patients with small cell lung cancer and other neuroendocrine cancers. Journal of Clinical Oncology. 2022;40(16_suppl):8566-.

8. Rudin CM, Liu SV, Soo RA, Lu S, Hong MH, Lee JS, et al. SKYSCRAPER-02: Tiragolumab in Combination With Atezolizumab Plus Chemotherapy in Untreated Extensive-Stage Small-Cell Lung Cancer. J Clin Oncol. 2024;42(3):324-35.

9. Uboha NV, Milhem MM, Kovacs C, Amin A, Magley A, Purkayastha DD, et al. Phase II study of spartalizumab (PDR001) and LAG525 in advanced solid tumors and hematologic malignancies. Journal of Clinical Oncology. 2019;37(15_suppl):2553-.

10. Powderly JD, Hamid O, Gutierrez ME, Balmanoukian AS, Janik J, Hoyle P, et al. 742P First-in-human phase I study of INCAGN02385, a LAG-3 monoclonal antibody antagonist in patients with advanced malignancies. Annals of Oncology. 2022;33:S883.

11. Edelman MJ, Dvorkin M, Laktionov K, Navarro A, Juan-Vidal O, Kozlov V, et al. Randomized phase 3 study of the anti-disialoganglioside antibody dinutuximab and irinotecan vs irinotecan or topotecan for second-line treatment of small cell lung cancer. Lung Cancer. 2022;166:135-42.

12. Chu Q, Leighl NB, Surmont V, van Herpen C, Sibille A, Markman B, et al. BMS-986012, an Anti-Fucosyl-GM1 Monoclonal Antibody as Monotherapy or in Combination With Nivolumab in Relapsed/Refractory SCLC: Results From a First-in-Human Phase 1/2 Study. JTO Clin Res Rep. 2022;3(11):100400.
